# Accessibility of Myofilament Cysteines and Effects on ATPase Depend on the Activation State during Exposure to Oxidants

**DOI:** 10.1371/journal.pone.0069110

**Published:** 2013-07-19

**Authors:** Sean M. Gross, Steven L. Lehman

**Affiliations:** Department of Integrative Biology, University of California, Berkeley, California, United States of America; Cinvestav-IPN, Mexico

## Abstract

Signaling by reactive oxygen species has emerged as a major physiological process. Due to its high metabolic rate, striated muscle is especially subject to oxidative stress, and there are multiple examples in cardiac and skeletal muscle where oxidative stress modulates contractile function. Here we assessed the potential of cysteine oxidation as a mechanism for modulating contractile function in skeletal and cardiac muscle. Analyzing the cysteine content of the myofilament proteins in striated muscle, we found that cysteine residues are relatively rare, but are very similar between different muscle types and different vertebrate species. To refine this list of cysteines to those that may modulate function, we estimated the accessibility of oxidants to cysteine residues using protein crystal structures, and then sharpened these estimates using fluorescent labeling of cysteines in cardiac and skeletal myofibrils. We demonstrate that cysteine accessibility to oxidants and ATPase rates depend on the contractile state in which preparations are exposed. Oxidant exposure of skeletal and cardiac myofibrils in relaxing solution exposes myosin cysteines not accessible in rigor solution, and these modifications correspond to a decrease in maximum ATPase. Oxidant exposure under rigor conditions produces modifications that increase basal ATPase and calcium sensitivity in ventricular myofibrils, but these effects were muted in fast twitch muscle. These experiments reveal how structural and sequence variations can lead to divergent effects from oxidants in different muscle types.

## Introduction

Cardiac and skeletal muscles are frequently exposed to concentrations of reactive oxygen species (ROS) that are detrimental to function [1]–[4]. In the heart this is most apparent during cardiac stunning when a burst of ROS depresses contractile performance and alters the calcium sensitivity of force production [1], [5]. Similarly, skeletal muscle is exposed to ROS during periods of activity and ischemia [2], [3]. In both tissues the impaired contractility is initially not due to reduced calcium concentrations, but instead is caused by the oxidation of amino acids found in the myofilament proteins [6]–[10].

Attempts to resolve which amino acids are critical to the oxidation response have focused on cysteines. Other amino acids including methionine and tyrosine can be modified by ROS, but the sulfhydryl group of cysteine is highly susceptible to modification [11]. Furthermore, actions that prevent or reverse cysteine oxidation can ameliorate the effects from oxidants in both the heart and skeletal muscle [12], [13]. Proteomic analyses of cardiac tissue have suggested that myosin, actin and tropomyosin (Tm) have cysteines that are critical to the effects from oxidants, but all myofilament protein in the heart except troponin T (TnT) and the regulatory light chain (RLC), which lack cysteines, can be modified by ROS [14]–[17]. Therefore, it is clear that cysteines are modified by ROS, but it is not always clear which cysteines are modified, or how function is altered by oxidation of a given cysteine.

ROS treatments can cause equivocal effects in different muscle types and even within a single muscle type [18]–[32]. For example, in studies on the heart some papers [9], [19] report a decrease in the calcium sensitivity, but others [18], [20]–[27] report no change or even an increase. This ambiguity has greatly complicated the task of linking an oxidized amino acid to a change in function. The Lamb laboratory advanced the field when they showed that slow twitch muscle responds differently from fast twitch muscle when cysteine oxidants were added while the muscle is contracting [29], implying that differences between oxidant responses may be based on differences between cysteines in fast and slow skeletal muscle, and suggesting that effects of oxidation may depend on the contractile state in which cysteines are exposed to oxidant [28]–[30].

Taking the lead of this groundbreaking paper [29], we set out to assess the potential of cysteine oxidation as a mechanism for modulating contractile function in skeletal and cardiac muscle.

First we catalogued the cysteine moieties in the primary sequences of the isoforms from many of the myofilament proteins found in the three striated muscle types (cardiac, skeletal slow-twitch, and skeletal fast-twitch). Aligning the amino acid sequences of isoforms of each myofilament protein, we found that most cysteines were conserved across vertebrate evolution, but some isoforms had cysteines not common with other isoforms. These differences may account for differences between ROS effects in different muscle types.

To discover which of the cysteines in an isoform of a myofilament protein were accessible to oxidants, we first scored the accessibility of each cysteine to solvent by finding its relative surface accessibility (RSA) in the tertiary structure. Only a fraction of cysteines in any myofilament protein had sizeable RSA. We then estimated the accessibility of myofilament proteins to oxidant in the myofilament lattice by labeling of myofibrils with fluorescent cysteine labels. Generally, the degree of labeling of a protein was proportional to the number of solvent-accessible cysteines, indicating that most solvent-accessible cysteines were also oxidant-accessible. However, the degree of labeling did depend on the presence of ATP, calcium ions, or both, suggesting that oxidant accessibility depended on the contractile state of the myofibril when it was labeled. This finding may account for differences between effects of ROS in experiments under different contractile conditions.

Finally, we measured ATPase of myofibrils from different muscle types at different calcium concentrations, after exposure to different oxidants under different contractile states, to test whether oxidation of cysteines accessible under different conditions modulated ATPase as predicted. We found that cysteine accessibility to oxidants and ATPase rates depend on the contractile state in which preparations are exposed. Oxidant exposure of skeletal and cardiac myofibrils in relaxing solution exposed myosin cysteines not accessible in rigor solution, and these modifications corresponded to a decrease in maximum ATPase, consistent with oxidation of cysteines in MHC. Oxidant exposure under rigor conditions produced modifications that increased basal ATPase and calcium sensitivity in ventricular myofibrils, consistent with modulation of activation of the thin filament. These effects were muted in fast twitch muscle. Our experiments reveal how structural and sequence variations lead to divergent effects from oxidants in different muscle types.

## Methods

### I. Cysteines in Primary Sequences and Molecular Structures

Myofilament protein sequences were accessed from the Uniprot database and aligned using the Clustal Omega program. Cysteine locations were analyzed using published crystal structures in the PDB database: chicken fast myosin: 2MYS, human cardiac troponin saturated with calcium (PDB: 1J1D and 1J1E), the calcium free and calcium saturated chicken fast troponin (PDB: 1YTZ and 1YV0), filamentous actin (PDB: 2ZWH), and Tm (PDB: 2B9C). To calculate the relative surface accessibility (RSA) of each cysteine, the ACC value from the DSSP output was divided by the total surface area of the residue [32].

### II. Experiments to Determine Cysteine Accessibility in Myofibrils

#### Animal, muscle and myofibril preparations

Ventricular, extensor digitorum longus (EDL), and soleus muscle samples were collected from Long-Evans rats. Muscle tissues were dissected within 20 minutes of euthanasia from cadavers of animals donated to us by other investigators, whose animal care and use protocol was approved by the UC Berkeley Animal Care and Use Committee. Following carbon dioxide exposure and cervical dislocation, the ventricles and EDL muscles were removed, minced, and homogenized in a solution similar in ionic strength and ion concentrations to intracellular conditions, but lacking ATP. This solution, referred to as rigor solution, contained 120 mmol/L potassium acetate, 50 mmol/L Hepes, 4 mmol/L magnesium chloride and 5 mmol/L EGTA with a pH of 7.0. The homogenates were repeatedly washed in rigor solution with 1% Triton - X 100 and spun at 16.1 relative centrifugal force (RCF) for 5 minutes. The supernatants containing mostly glycolytic and membranous proteins were discarded. After repeated washing the enriched myofibril preparations were stored at −20°C in a solution of 50% glycerol and 50% rigor until future use.

#### Fluorescent labels

To assess different cysteine-specific fluorescent reagents skeletal myofibrils were treated with either tetramethylrhodamine-5-maleimide (TMR), bodipy FL maleimide (BFM), or 5-(iodoacetamideo)fluorescein (IAF) for five minutes. TMR has a mass of 481 Da and contains one positive and one negative charged group. BFM has a mass of 414 Da and does not contain charged groups. IAF has a mass of 515 and contains two negative and one positive charged groups. The reaction was quenched by adding 10 mmol/L DTT. To assess the sensitivity of myofilament cysteines to modification, myofibrils were treated with 10, 25, 50, 100, 250, 500 and 750 µmol/L of TMR in rigor buffer. The reaction of TMR with myofibril cysteines was quenched after five minutes by the addition of 10 mmol/L DTT. Each fluorescent reagent was prepared as a 10 mmol/L stock in dimethylformamide (DMF).

To analyze TnC cysteine accessibility, we varied the concentration of calcium and chelating agents in the SDS-PAGE sample buffer. EGTA chelates calcium bound to TnC and causes TnC to focus at a higher relative molecular weight [33]. The effect of calcium on TnC cysteine accessibility was tested by treating myofibrils with 100 µmol/L TMR in rigor solutions that contained [calcium] from pCa 5.0 to 8.5. The reaction was quenched after five minutes with 10 mmol/L DTT.

To label cysteines that were accessible in relaxing solutions, myofibrils reduced with 10 mmol/L DTT, were treated in relaxing solution with 250 µmol/L TMR, and the reaction was quenched after either 5 minutes (submaximal labeling) or 60 minutes (saturation labeling) with 10 mmol/L DTT. To label the total population of cysteines, and not just those accessible in their native environment, myofibrils were denatured in a solution containing 8 mol/L urea, 2% SDS, 25% glycerol, 125 mmol/L Tris, pH 6.8. Denatured myofibrils were then labeled with 250 µmol/L TMR. The remaining TMR was quenched after 60 minutes by the addition of 10 mmol/L DTT. We estimated the ratio of moles of TMR to moles of myofibril thiols at 10∶1, assuming the content of myofibril thiols was ∼68 nmol/mg myofibril protein [24].

#### SDS PAGE Electrophoresis

Following TMR treatments, myofibril protein samples were analyzed using 5% separating, 12.5% resolving SDS PAGE gels. After electrophoretic focusing in the gels, proteins with incorporated TMR were imaged using a Typhoon scanner and 532 nm excitation and 580 nm emission wavelengths. Gel samples labeled with either bodipy FL maleimide (BFM), or 5-(iodoacetamideo)fluorescein (IAF) were scanned at 488 nm excitation and 520 nm emission wavelengths. After the TMR (or BFM and IAF) scan, gels were fixed for 30 minutes (50% methanol and 10% acetic acid), washed in water, and stained overnight with Sypro Ruby to visualize every protein independent of its cysteine content. Gels stained overnight were placed in de-stain solution (10% methanol and 7% acetic acid) for 30 minutes, washed twice with water for five minutes, and scanned using 488 nm excitation and 610 nm emission wavelengths.

### III. Experiments to Test Myofibril ATPase after Oxidant Treatments

Myofibril ATPase was assessed using a malachite green microplate protocol that measures the amount of phosphate produced from ATP hydrolysis [34]. Myofibril cysteines were first reduced in rigor solution with 10 mmol/L dithiotheritol (DTT) for at least 30 minutes and then washed twice in rigor solution to remove residual DTT. The myofibrils were then treated with oxidant (see oxidant treatments below) at room temperature. Following treatment, myofibrils were washed twice in rigor solution to remove all traces of oxidant and treatment solution. Myofibrils were then re-suspended in rigor solution with 1mg/ml bovine serum albumin (BSA) and divided equally into 10 tubes. To initiate the ATPase reaction, calcium solutions (pCa 5 - 8.5) with 4 mmol/L ATP (final concentration 3 mmol/L) were added to the myofibrils. The calcium solutions were prepared by mixing the relaxing (pCa 8.5) and activating solutions (pCa 5.0) to achieve the desired calcium concentration based on calculations from the MaxChelator online program. The ATPase reaction was stopped after five minutes with the addition of perchloric acid and malachite green solution. Fifteen minutes after the addition of malachite green the absorbance was measured at 650 nm. All samples were measured in duplicate.

#### Myofibril oxidant treatments

For ATPase assays myofibrils were treated with several cysteine-specific reagents that differ in their type of cysteine modification. 2-2′ dithiodipyridine (DTDP), an aromatic disulfide that reacts with protein thiols to form a mixed disulfide, was prepared as a 20 mmol/L stock solution in 50% ethanol, and myofibrils were treated for five minutes with 75 µmol/L or 150 µmol/L (15 or 30 nmoles). N-Ethylmaleimide (NEM), which irreversibly alkylates thiols, was made as a 20 mmol/L stock solution in rigor buffer, and myofibrils were treated for five minutes with 75 µmol/L. S-Nitroso-N-acetyl-DL-penicillamine (SNAP), a nitric oxide (NO) donor, was prepared immediately prior to each treatment as a 40 mmol/L stock solution in rigor buffer and used at 2 mmol/L SNAP. This concentration of SNAP is expected to release ∼2 µmol/L of NO [35]. Samples treated with SNAP were protected from light to prevent reversal of protein nitrosylation.

### IV. Quantification and Statistics

Statistical significance was assessed through use of two-tailed student’s t-tests using Prism 5.0 software. A P-value <0.05 was considered significant. Band fluorescence in SDS PAGE gels was measured by quantifying the pixel density in a box containing the band of interest and subtracting the background pixel density. The maximal and basal ATPase rates, and the pCa_50_, and *n*_H_ were determined by fitting ATPase rates as a function of calcium concentrations using a Hill equation and Prism 5.0 software. Unless otherwise noted values are shown as mean ± SEM.

## Results

### Cysteines in Isoforms of Myofilament Proteins

**Figure 1:**
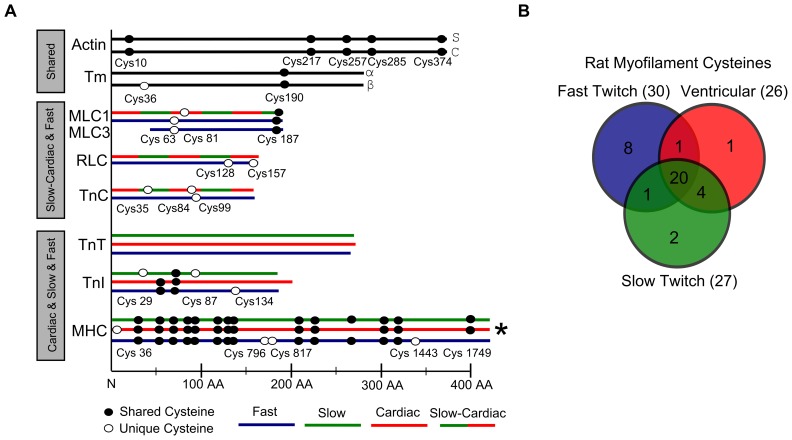
Cysteines in isoforms of myofilament proteins. A. Positions of cysteines in primary sequences of myofilament proteins from rat skeletal and cardiac muscles. Myofilament protein isoforms are represented by horizontal lines whose colors denote different muscle types: Actin has skeletal and cardiac isoforms; Tm is a dimer with alpha and beta isoforms; other myofilament proteins have fast-twitch (blue), slow-twitch (green), cardiac (red) and slow-twitch/cardiac (green/red) isoforms. Black circles denote cysteines conserved in at least two isoforms; white circles denote cysteines unique to a single isoform. Cysteine numbering is based on the rat primary sequences. The scale on the x-axis indicates approximate number of amino acids from the N-terminus for all myofilament proteins except *MHC, whose ∼1939 AA sequence had to be condensed to fit. B. Venn diagram showing number of cysteines expressed in cardiac, fast, or slow fiber types. Most myofilament cysteines are common to all three types, many are common to ventricular and slow skeletal but not fast skeletal muscle, and several are unique to fast skeletal muscle.

Striated muscles of different types have many myofilament proteins in common. Cardiac, slow-twitch and fast-twitch skeletal muscles often have distinct isoforms of myofilament proteins that have evolved to have somewhat different primary sequences. We compared the presence of cysteines between the different rat myofilament proteins isoforms (Figure 1A). Actin has cardiac and skeletal isoforms, both containing the same five cysteines. In skeletal muscle two Tm isoforms (alpha and beta) are expressed, whereas cardiac muscle predominantly expresses the alpha isoform. The alpha and beta isoforms have one cysteine in common (Cys 190), and the beta isoform has a second cysteine (Cys 36). Myosin essential light chain (MLC1) has one isoform that is co-expressed in cardiac and slow twitch muscle (cMLC1), and a second isoform that is expressed in fast-twitch muscle (fMLC1). The fast-twitch isoform is alternatively spliced to produce two protein products (MLC1 and MLC3), which differ in size due to a truncation at the N-terminus of MLC3. MLC1 has two cysteines in the cardiac and fast isoforms. Cys 187 is common to cMLC1 and fMLC1; the other MLC1 cysteine differs between cardiac and fast (cMLC1 Cys 81, fMLC1 Cys 63). Both cysteines present in the fast isoform of MLC1 are also found in MLC3. RLC and TnC have one isoform specific to fast twitch muscle and one isoform co-expressed in cardiac and slow twitch muscles. Cardiac RLC lacks cysteines; fast RLC has two cysteines. The cardiac TnC isoform has two cysteines (Cys 35 and Cys 84); the fast isoform has one cysteine (Cys 99). TnT, TnI and MHC have unique isoforms for cardiac, slow, and fast twitch muscles. TnT lacks cysteines in all three isoforms. TnI isoforms have either two or three cysteines, but placement between the isoforms is not fully conserved. There are multiple MHC isoforms expressed in striated muscle. The predominant isoforms expressed in adult rat cardiac, slow, and fast twitch muscles are Myh6, Myh7, and Myh4 respectively. In these three MHC isoforms, 12 cysteines have the same positions. There are five cysteine differences between slow and fast MHC, four differences between fast and cardiac, and two differences between cardiac and slow.

To make comparisons between muscle types, we compiled the myofilament cysteines that were conserved across isoforms and those that were unique to a single isoform. Slow twitch myofilament proteins contain 27 cysteines (3,582 total amino acids), ventricular myofilament proteins have 26 cysteines (3,634 total amino acids) and fast twitch muscle contains 30 cysteines (3,558 total amino acids) (Fig. 1B). The average cysteine frequency in the myofilament proteins is 0.8%, which is less then the frequency in the human proteome (2.3%) [36]. The modification of cysteines common between different muscle types should cause similar effects in all muscle types. Of the 37 total cysteines, 20 are shared between all fiber types (Fig. 1B). Cardiac and slow twitch myofilament proteins share 24 cysteines, and contain one and two unique cysteines respectively.

### Cysteines in Myofilament Proteins are Well Conserved in Vertebrates

**Figure 2:**
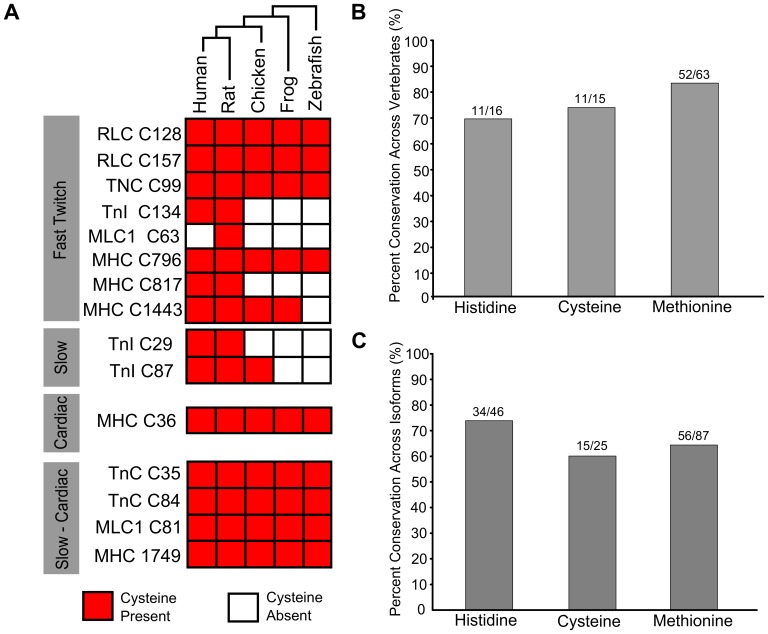
Cysteines unique to an isoform are well conserved in vertebrate evolution. A. Presence of unique cysteines in the rat isoforms, compared to their presence in human, rat chicken frog and zebrafish isoforms. Each row represents a cysteine that is found in only one of the isoforms of a rat myofilament protein. Columns represent species (evolutionary relationships are displayed at top). Red boxes indicate that the cysteine in the rat isoform is present in the isoform of the other species. B. Percent of histidine, cysteine, and methionine residues conserved across five vertebrate species (human, rat, chicken, frog, zebrafish) in fast and slow isoforms of TnI, TnC, RLC, and MLC. C. Percent of methionine, histidine and cysteine residues common to fast and slow isoforms of rat MHC, TnI, TnC, RLC and MLC relative to the total number in the fast isoform.

The conservation of cysteines between muscle types led us to ask when during vertebrate evolution each cysteine specific to one isoform emerged. Analysis of fish, frog, chicken, rat and human amino acid sequences revealed that nearly all isoform specific cysteines emerged early in vertebrate evolution, prior to the divergence of fish and amphibians (Fig. 2A). Among the isoform specific cysteines that emerged more recently, two were analyzed in further detail. Cysteine 134 of fTnI is absent from fish, frog, chicken, quail and turkey sequences, but is present in the zebra finch and chameleon sequences, indicating that not all birds and reptiles lack this site (Fig. S1B). Cys 63 of rat fast MLC1 is unusual across vertebrate evolution since it is expressed in some slow and some fast isoforms. The cysteine is found in the human ventricular isoform and rodent fast isoforms, but is absent from rodent ventricular isoforms and the human fast isoform. Analysis of additional vertebrate species shows that this cysteine is found in slow frog isoforms, but not in fast isoforms of frog, bird, or fish sequences (Supplementary Fig. S2).

To test whether cysteines have been more conserved compared to other amino acids expressed at similar frequencies, we examined the number of shared cysteines, methionines, and histidines. In the human codon usage database methionine is present at a frequency of 2.2%, cysteine at 2.3% and histidine 2.6%. In fast and slow isoforms of four myofilament proteins (TnI, TnC, RLC, and MLC1) across five vertebrate species (human, rat, chicken, frog, zebrafish) (see Fig. S1A for an example), we found that 68.7% (11/16) of histidines, 82.5% (52/63) of methionines, and 73% (11/15) of cysteines in the human isoforms were conserved across the five species analyzed (Fig. 2B). As a second comparison, we analyzed the fraction of shared methionines, histidines and cysteines between the fast and slow isoforms of MHC, TnI, TnC, RLC and MLC1 compared to the total number in the fast isoform. Between isoforms histidine was most conserved 73.9% (34/46), followed by methionine 64.4% (56/87), and cysteine 60% (15/25) (Fig. 2C). Thus, there is no evidence that myofilament cysteines have been more conserved compared to other similarly expressed amino acids either within a muscle type or between muscle types.

### Structures Predict Accessibility of Myofilament Cysteines to Solvent

**Figure 3:**
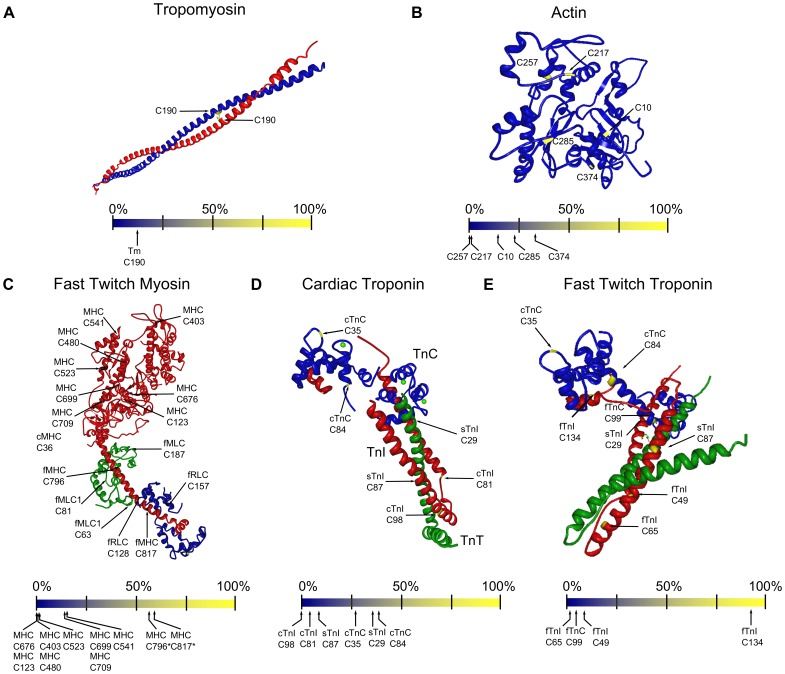
Structures predict accessibility of myofilament cysteines to solvent. A. Location of cysteines in a tropomyosin heterodimer. The structure shown is the alpha homodimer (PDB structure: 2B9C), with the blue strand labeled to indicate locations of cysteines in the alpha isoform. B. Cysteine locations in one monomer of F-actin (PDB: 2ZWH) C. Location of cysteines in the fast muscle isoform of MHC (PDB: 2MYS). Cysteine numbering in MHC is based on rat MYH4 D,E. Locations of cysteines in cardiac (D) and fast (E) skeletal isoforms of troponin (TnC:Blue, TnI:Red, TnT:Green) (Cardiac troponin is PDB:1J1E; Fast skeletal troponin is PDB: 1YTZ). For comparison, cysteines in slow (s), cardiac (c) and fast (f) isoforms are labeled in each structure. Relative surface accessibility (RSA) values of cysteines are shown below the structures.

Protein crystal structures provide insight into the accessibility of cysteines by oxidants, and the capacity of specific cysteines to alter function. Using the chicken fast MHC (PDB: 2MYS), human cardiac troponin saturated with calcium (PDB: 1J1D and 1J1E), the calcium free and calcium saturated chicken fast troponin structures (PDB: 1YTZ and 1YV0), filamentous actin (PDB: 2ZWH), and Tm (PDB: 2B9C) we calculated the relative surface accessibility (RSA) of the myofilament cysteines (Fig. 3).

Tm is a long dimer (Fig. 3A) composed of two alpha helical chains. In the Tm dimer Cys 190 of each monomer is aligned, partially accessible based on RSA analyses, and thus capable of forming a disulfide bond upon cysteine oxidation.

Each actin monomer has five cysteines, and at least two are likely to be accessible based on RSA analyses (Figure 3B). Cysteine 374, the second-to-last residue in the C-terminal helix is highly accessible to solvent based on our RSA calculations. Similarly, the nearby cysteine in the N-terminus of actin (C10) is also likely to be accessible. C285 may be partially accessible based on its RSA value, whereas the remaining two cysteines in actin (C217 and C257) have low accessibility.

The S1 fragment of fast MHC has 10 cysteines (Fig. 3C and Fig. S3). Five cysteines (C123, 403, 480, 523, 676) have low predicted accessibility to solvent and three cysteines (C541, C699, C709) have RSA numbers in the range of 20%. C541 is in the lower 50 K subdomain, and C709 and C699, also known as SH1 and SH2 are positioned near the ATP binding site. Two cysteines, C796 and C817, are located in the neck region and are unique to fast isoforms (Fig. S3). C796 is located in an MLC binding region, and C817 is located in an RLC binding region. In the absence of light chains these cysteines are highly accessible based on their RSA values (Figure 3C). Two cysteines, C1443 (fast) and C1749 (slow and cardiac), are located in the tail domain, and are unlikely to modulate myosin II function.

The myosin heavy chains expressed in striated muscle are part of an ancient lineage that has been superbly conserved across evolution. Since two distantly related species are unlikely to use oxidative modulation similarly, it is more likely that common cysteines stayed in place for structural reasons. Comparing the slime mold Dictyostelium discoideum Dd Myo to Rat MYH4 reveals that four cysteines (C480, 676, 699, and 1415) have been conserved between the two distantly related species and thus are unlikely to be responsible for oxidative modulation.

In the myosin structure Cys 63 of the fast isoform of MLC1 (Fig. 3C) is located in a loop adjacent to RLC and is two amino acids away from an MLC1 phosphorylation site [37]. The expression of this cysteine is variable throughout vertebrate evolution and between isoforms (Fig. S2). Cys 81 of cMLC1 is unique to the cardiac isoform and is located near the neck region of MHC and Cys 796. The last MLC1 cysteine (C187), conserved in both the cardiac and fast isoforms, is located near the head region of MHC. The fast isoform of RLC contains two cysteines; Cys 128 is located in a loop near MLC1 Cys 63 while RLC Cys 157 is located on the opposite side of the protein close to the MHC neck. The ventricular isoform of RLC lacks cysteines.

In the troponin complex there are eight different cysteines between the muscle types. The cysteine in fast TnC (Cys 99) is positioned between TnI and TnT and has a low RSA value, which suggests inaccessibility to oxidants (Fig. 3E). Cardiac TnC (Fig. 3D), co-expressed in slow and cardiac muscle contains two cysteines (Cys 35 and Cys 84) located on opposite sides of the regulatory N-domain that have relatively high RSA values.

The cysteine unique to fTnI (Cys134) is located in a loop adjacent to the switch segment and is highly accessible in the calcium saturated troponin structure (Fig. 3E). The two other cysteines in fast TnI have low RSA values in the calcium-saturated structure, but their RSA values are marginally higher in the calcium free state and may become partially accessible. These two fTnI cysteines are conserved in the cardiac isoform and are similarly predicted to be inaccessible in the calcium saturated cardiac complex. Slow TnI contains two additional cysteines not found in the other two isoforms. Cys 29 is located in a coil near Cys 99 of fast TnC, and Cys 87 is located in a coil adjacent to TnT. There is not a structure for slow TnI, but using the cardiac structure as a guide, Cys 29 would be exposed whereas Cys 87 would be inaccessible.

In the troponin complex, we saw no evidence for closely aligned cysteines, unless isoforms from different fiber types were mixed. Mixing fTnC with sTnI results in Cys 99 of fTnC being ∼4 angstroms apart from Cys 87 of sTnI and thus capable of forming a disulfide (Fig. 3E).

### II. Results of Experiments to Measure Cysteine Accessibiltiy

Fluorescent probes identify the subset of cysteines accessible in the myofilament lattice. RSA analyses predicted that not all cysteines are accessible when isolated outside of the protein-protein interactions that shape the myofilament lattice. To assess cysteine accessibility in the myofilament lattice, we used cysteine-specific fluorescent probes to label and quantify cysteine accessibility in myofibrils [38]. We began by testing three fluorescent probes that differed in charge, structure, and excitation-emission wavelengths. Two probes, TMR and BFM, had a cysteine-specific maleimide moiety, analogous to the non-fluorescent N-ethyl maleimide that has been extensively used for cysteine analyses. A third reagent, IAF, has a different reactive group and has also been used frequently for cysteine analyses.

**Figure 4:**
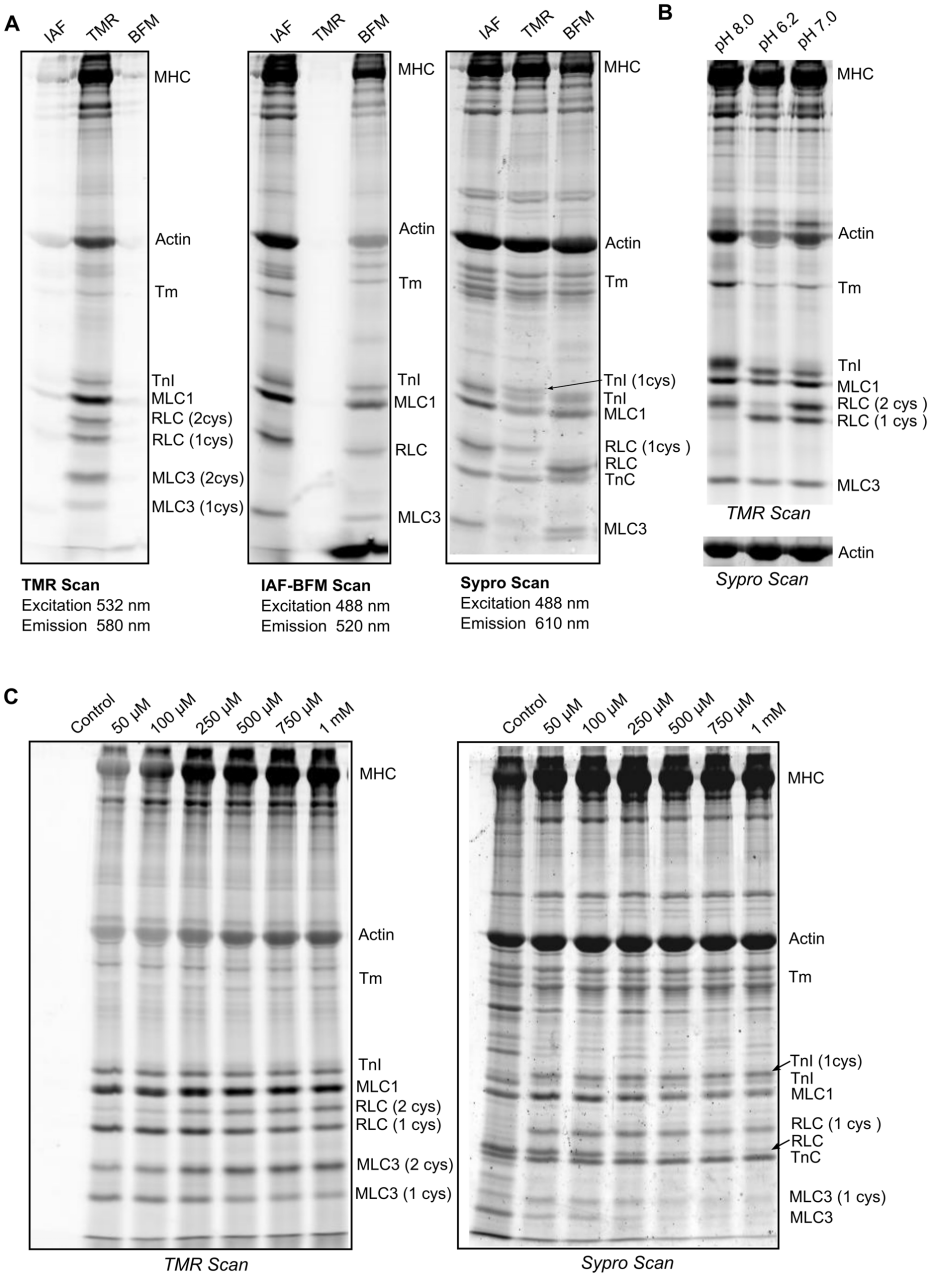
Fluorescent labels identify cysteines as accessible to solvent in the filament lattice. A. Rat EDL myofibrils were treated with one of three cysteine-specific fluorescent reagents, TMR, BFM or IAF. The myofibril proteins were then denatured and separated on an SDS PAGE gel. The gel was then scanned twice, at excitation and emission wavelengths appropriate to TMR or IAF and BFM. The gel was next stained overnight with Sypro, and scanned at excitation and emission wavelengths appropriate to Sypro. B. TMR scan of a SDS-PAGE gel of myofibrils treated with 100 uM TMR for 5 minutes at pH 6.2, 7.0, or 8.0. The actin bands from the three lanes stained with Sypro are displayed below the TMR scan as a loading control. C. Left: SDS-PAGE gel of rat EDL myofibrils treated for 5 minutes with a range of TMR concentrations (0–1 mM) and scanned using the TMR excitation emission settings. Right: The same gel was then stained with Sypro and scanned at Sypro frequencies.

Myofibrils were treated separately with each fluorescent probe. The proteins were then denatured and electrophoresed on SDS-PAGE gels (Fig. 4A). Each was scanned at excitation and emission wavelengths appropriate to the probe to visualize proteins with cysteine-bound probes, and then later at the Sypro excitation and emission wavelengths to visualize the total protein population. It is important to note that the intensity of bands will differ between the cysteine bound probes and the total protein stain, as Sypro depends on the amount of protein and its size, and the cysteine-bound probes depend on the amount of protein and the number of modified cysteines.

Treatment of fast skeletal myofibrils with the three fluorescent cysteine reagents revealed that each reagent labeled cysteine-containing proteins based on the fluorescence in the TMR and IAF-BMF scans (Fig. 4A). The three reagents produced different banding patterns in the lower molecular weight proteins (TnI, MLC1, RLC and MLC3). Comparison of the positions of the TnI, RLC, and MLC3 bands between the TMR and Sypro scans suggested this difference was due to the TMR probe adding a mass to the protein, based on the number of cysteines modified: the TMR-labeled proteins ran slower on the gels. The shift of the MLC3 band labeled with BMF suggested that this probe also altered electrophoretic mobility. Based on the high rate of reaction, bright fluorescence, clear bands, and vertical mass shift, we chose TMR to characterize further as a tool for identifying accessible cysteines.

The reactivity of a cysteine with oxidants is dependent on its sulfhydryl group being in the thiolate form. The more basic the solution pH, the greater fraction of time the sulfhydryl group is present in the thiolate form. To assess whether TMR was reacting with the thiolate form, we briefly treated myofibrils with TMR in solutions with different pH values. Treatment of myofibrils with TMR in solutions of increasing pH led to increasing fluorescence intensity (Fig. 4B). For example, intensities of actin, MLC1 and RLC at pH 6.2 and pH 7.0 averaged 57% and 76% of the intensities at pH 8.0. The shift in RLC bands, from a single band at pH 6.2, through this band and a slower one at pH7.0 to only the slower band at pH 8.0 was especially strong evidence that the reactivity of TMR with myofilament cysteines depended on the sulfhydryl group being in the thiolate form.

To assess whether the binding of TMR to cysteine led to a mobility shift that was detectable in the SDS-PAGE gels, we treated myofibrils with increasing doses of TMR (Fig. 4C and Figure S4). We found that as the dose of TMR increased, there was a progressive slowing of the low-molecular-weight proteins, and increased fluorescence of the bands representing RLC and MLC3 with both labeled cysteines (each of these proteins have two cysteines). Note that the singly-labeled bands decreased fluorescence intensity correspondingly. The presence of fTnC in the Sypro, but not in the TMR scan indicates its cysteine is inaccessible. These changes were consistent across experiments (Fig. 4C and Figure S4). A TMR dose response for cardiac muscle, which showed a similar increase in band intensity as the dose of TMR increased, is shown in Fig. S5. In summary, TMR could identify proteins with accessible cysteines in the myofilament lattice, and for small molecular weight proteins could provide a measure for the number and fraction of modified cysteines.

**Figure 5:**
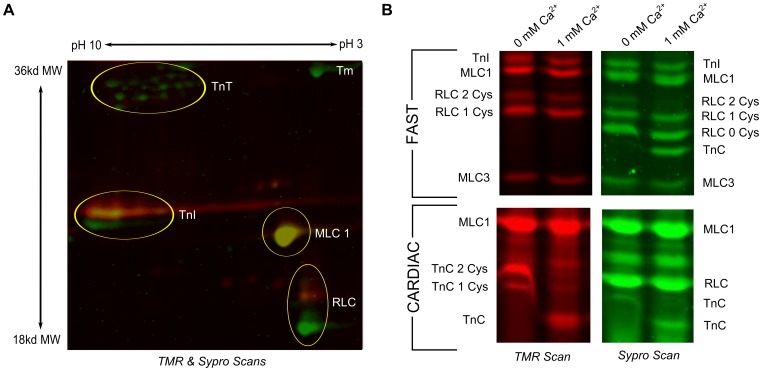
Confirmation of accessible cysteines in fast skeletal muscle. A. 2D NEPHGE gel (pH 3–10) of rat EDL myofibrils with the TMR (red) and Sypro (green) scans superimposed. Yellow indicates overlap of TMR and Sypro fluorescence signals. B. TMR (left, red) and Sypro (right, green) scans from myofibrils (EDL top, Ventricles bottom) labeled in TMR then run in SDS-PAGE gels in sample buffer without added calcium (lane 1) or with 1 mM added calcium (lane 2).

To identify additional accessible cysteines and to better distinguish between fTnI and fMLC1, which have similar molecular weights, we separated proteins based on both their isoelectric point and molecular weight using 2D IEF-SDS PAGE gels, and then superimposed the TMR and Sypro scans (Fig. 5A). These gels confirmed that the fast isoform fTnI had at least one reactive cysteine, based on the TMR fluorescence (red/yellow) and the increase in molecular weight from the bound TMR. Additionally, the red/yellow fluorescence for MLC1 and RLC confirmed these proteins had accessible cysteines. It is important to note that TMR labeling of MLC1 did not change its electrophoretic mobility. These measurements in the myofilament lattice are in concordance with the RSA values for TnI and TnC from the crystal structures. TnT, which lacks a cysteine in its primary sequence, was not labeled with the red TMR indicating TMR did not react non-specifically with other amino acids (Fig. 5A).

To confirm the identification and cysteine accessibilities of TnC and RLC, which also have similar molecular weights, we compared two TMR-labeled samples that were run in 1D SDS-PAGE gels (Fig. 5B). The samples were identical except one sample contained 1 mM calcium in the SDS-PAGE sample buffer, and the other sample lacked added calcium. We know from the literature that TnC focuses at a molecular weight below RLC in the presence of calcium and with RLC in the absence of RLC [33]. Our Sypro scans confirmed these findings, for fast and cardiac muscles, with the fTnC band merged with the fRLC 0 band, and the cTnC band merged with the cRLC band in the absence of calcium (Fig. 5B). Furthermore, in the Sypro scan we observed three RLC bands in fast muscle but only one RLC band in cardiac muscle. We know from primary sequence data that fRLC has two cysteines, and cRLC has no cysteines. We interpret the three RLC bands in fast muscle as fRLC with 0,1 and 2 cysteines labeled. From primary sequences, fTnC has one cysteine and cTnC has two cysteines. In the TMR scan we find two cTnC bands in the absence of calcium, interpreted as singly- and doubly-labeled cTnC, consistent with our accessibility RSA estimate from structure (Fig. 3). fTnC has no cysteines, and no TnC bands are visible in the TMR scan of the fast samples. Thus, the one- and two-dimensional gels confirmed that fMLC1, fMLC3, fRLC and fTnI, but not fTnC had cysteines that could be modified in fast skeletal muscle. In cardiac muscle cTnC and cMLC1 both contained accessible cysteines. The biochemical data (Figs. 4 and 5) and the structural data (Fig. 3) are in accord.

### Direct Comparisons of Cysteine Accessibilities between different Muscles and Fiber types

**Figure 6:**
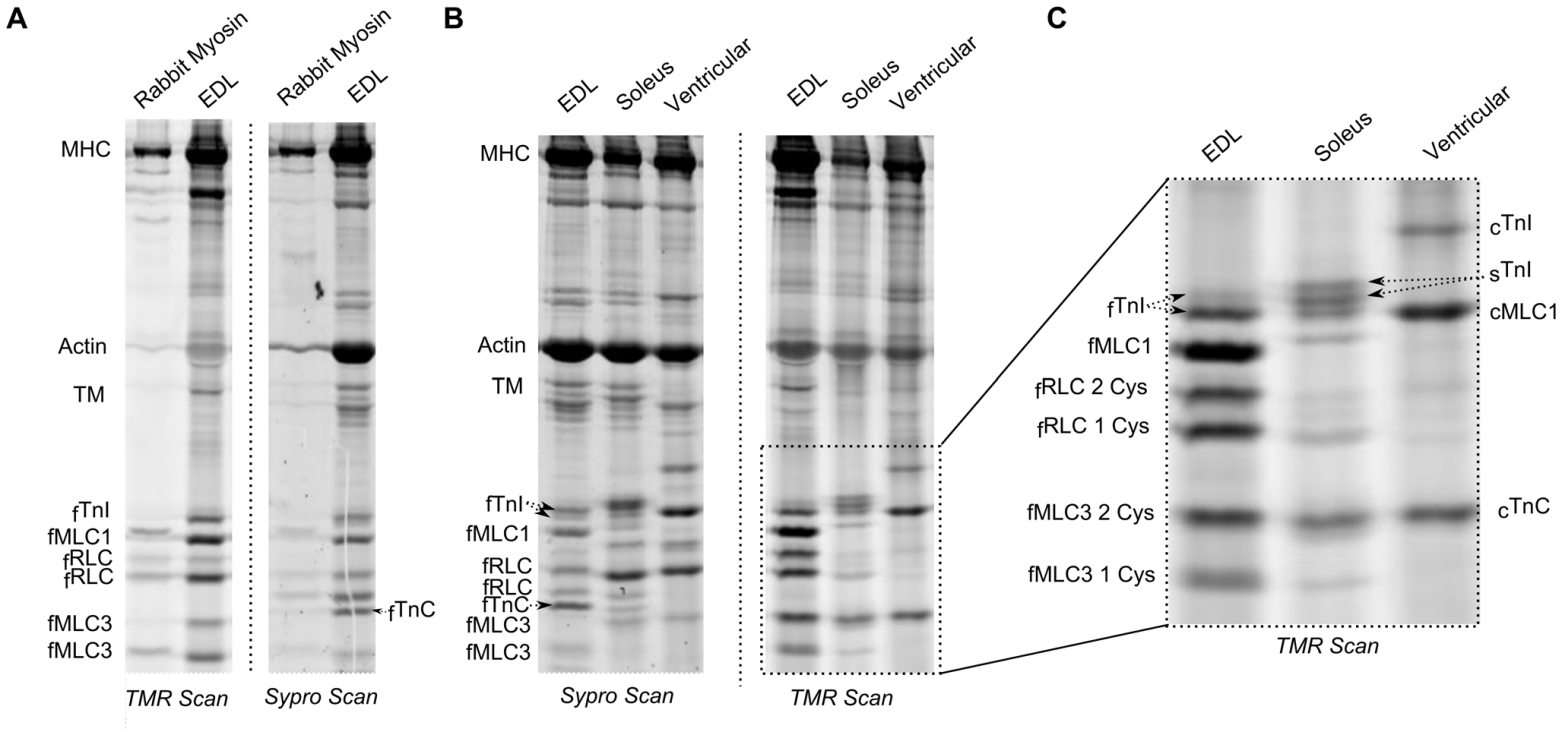
Direct comparisons between cysteine accessibilities in myofilament proteins from different muscles. A. SDS-PAGE gels of purified rabbit myosin heavy chain and light chains (MHC, RLC, MLC1, MLC3) and rat EDL myofibrils, treated with TMR and run in adjacent lanes. TMR and Sypro scans (left and right) indicate that cysteines in fRLC, fMLC1 and fMLC3 are accessible, but cysteines in fTnC are not. B,C. SDS-PAGE gel shows the Sypro and TMR scans of fast (rat EDL), slow (rat soleus), and cardiac muscle (rat ventricle), showing accessibility of the low molecular weight proteins from each fiber type.

To further characterize cysteine accessibility, we compared TMR-treated purified myosin (containing MHC, RLC, MLC1/3) from rabbit fast twitch muscle with myofibrils from rat EDL (Fig. 6A). In the TMR scan, the fRLC, fMLC1 and fMLC3 bands in the myosin protein lane match bands in the EDL lane, confirming their identities as myosin light chains. The rabbit fMLC1/3 sequence contains a single cysteine, while the rat sequence contains two cysteines (Fig. 1); correspondingly, a single band is present in the rabbit myosin lane, whereas two bands are present in the rat EDL lane, indicating that both rat fMLC3 cysteines are accessible. The two bands for fRLC in the rabbit myosin and rat TMR EDL lanes indicate that both cysteines are accessible. A fTnC band is present in the rat EDL lane of the Sypro scan but not in the TMR scan – fTnC is inaccessible.

Next we compared accessibilities of cysteines in fast myofibrils (EDL) to cysteines in slow (soleus) and ventricular muscles (Fig. 6B and C). It is important to note that EDL and especially soleus are not pure muscle types and instead contain a fraction of slow and fast isoforms respectively. As a consequence faint bands for fast isoforms can be seen in the soleus lane. In the ventricular lane we observe cysteine accessibilities for cTnC, cMLC1 and cTnI (cTnC focuses at the same MW as doubly-labeled fMLC3; cMLC1 has a molecular weight close to fTnI). cRLC, which lacks cysteines, is present in the Sypro scan but not in the TMR scan. Isoforms of slow MLC1, RLC and TnC are identical to the isoforms of cardiac MLC1, RLC and TnC, but slow isoforms of TnT and TnI are different from the cardiac isoforms (Fig. 1). No isoform of TnT has a cysteine; slow TnI has three cysteines and cardiac TnI has two. Based on the two unique bands above cMLC1 in the soleus lane, and the similar molecular weights of cMLC1 and sTnI, it appears sTnI has two accessible cysteines.

### Comparison of Accessible Cysteines with Total Cysteines

**Figure 7:**
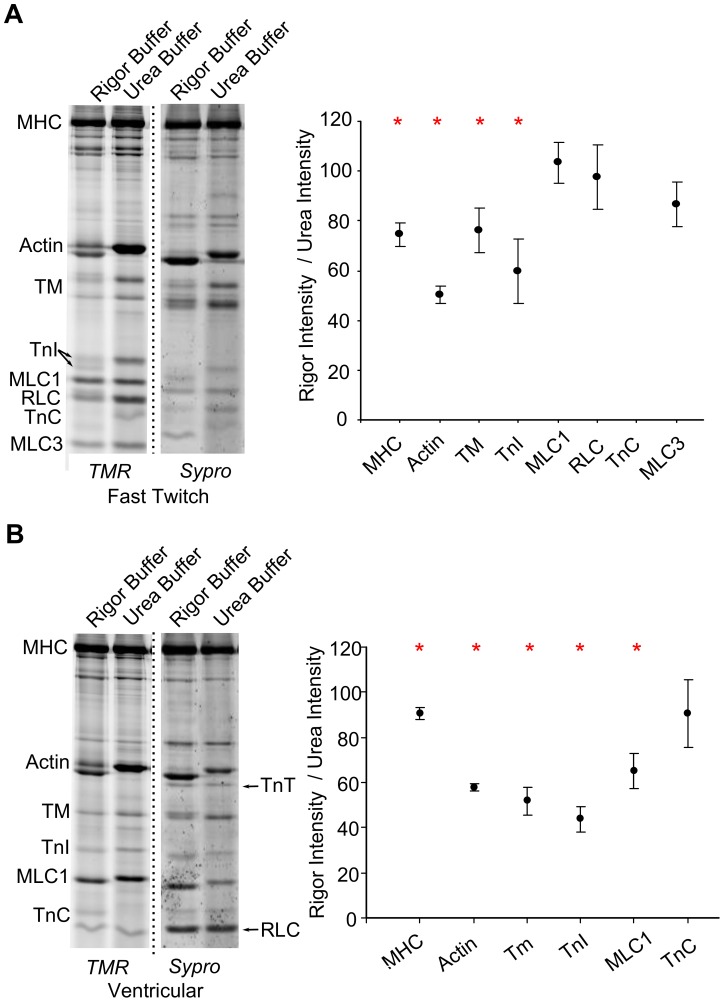
Ventricular and EDL myofibrils contain a population of cysteines that are inaccessible to oxidants. TMR and Sypro scans from (A) EDL and (B) ventricular myofibrils treated with TMR in either rigor buffer or 8 M urea buffer to compare the accessibility of cysteines in the myofilament lattice to accessibility in denatured proteins. The ratio of fluorescence intensities in rigor to urea buffers for each myofilament protein is plotted on the right (mean ± SEM, *n* = 5). Red stars indicate that the TMR fluorescence intensities under the two treatments were significantly different (p<0.05).

To observe how the population of cysteines accessible to solvent in rigor solution compared to the total cysteine population, we labeled one sample of myofibrils with TMR in rigor solution (to label accessible cysteines), and labeled a second sample after treating the myofibrils with urea to denature the proteins (to label total cysteines). We then focused both samples in SDS-PAGE gels and analyzed TMR fluorescence (Fig. 7).

Fast-twitch skeletal MHC, actin, Tm and TnI had significantly greater fluorescence after urea treatment, indicating that these myofilament proteins had inaccessible cysteines (Fig. 7A). Likewise, fast-twitch TnC did not fluoresce in rigor solution unless pre-treated with urea. The fluorescence intensities of MLC1, MLC3 and RLC were not significantly different between the two treatments, indicating that these myofilament proteins have no inaccessible cysteines.

Ventricular MHC, actin, Tm, TnI and MLC1 had significantly greater fluorescence after urea treatment, indicating that these myofilament proteins had inaccessible cysteines (Fig. 7B). Actin, TnI and MLC1 ran slower after urea treatment, further indicating that the TMR probe had bound additional cysteines to these lower molecular weight proteins. There was no significant difference between fluorescence intensities of TnC in the two samples, and TnT and RLC, which lack cysteines, were absent in the TMR scans but present in the Sypro scans.

### Contractile State Affects Cysteine Accessibility

**Figure 8:**
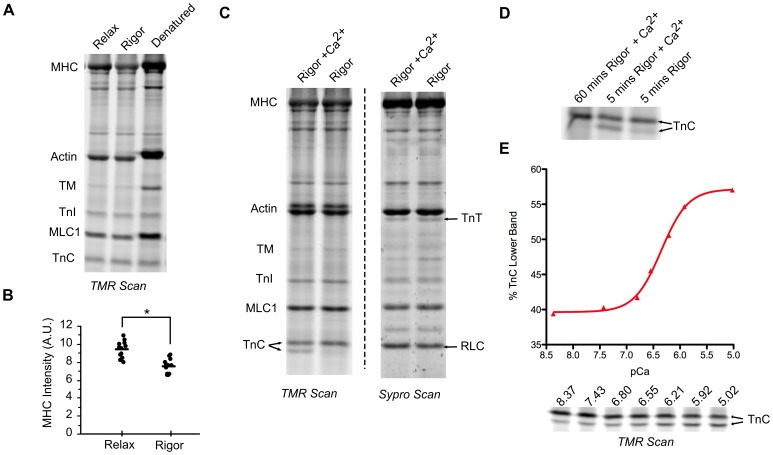
The TMR fluorescence of several myofilament proteins is sensitive to the contractile state. A. TMR scan of ventricular myofibrils briefly treated with TMR under rigor, relaxing or denaturing conditions. B. Dot plot of MHC TMR fluorescence (arbitrary units = AU) from relaxing and rigor conditions. * indicates p<0.05 C. TMR and Sypro scans from myofibrils labeled in rigor solution+Ca^2+^, or rigor solution for five minutes (100 µmol/L TMR). D. TMR scan of TnC labeled in rigor+calcium for 60 minutes, rigor+calcium for 5 minutes, and rigor without added calcium for 5 minutes (100µmol/L TMR). E. Graph and TMR fluorescence of the fraction of TnC fluorescence in the lower of the two TnC bands, over a range of [Ca^2+^] (rigor +calcium, 100 µmol/L TMR, 5 minutes).

Not all myofilament cysteines were accessible in rigor solutions (Fig. 7). We therefore hypothesized that several myofilament proteins contain cysteines whose accessibility depended on the contractile state. To measure the accessibility of cysteines in different contractile states, we compared the gel fluorescence from myofibrils labeled with 100 µmol/L TMR in rigor solution (no ATP) where myosin heads were presumably attached to actin in a rigor bond, and in relaxing solution (3 mmol/L ATP) where myosin heads were presumably not strongly attached to actin. In the SDS PAGE gels, the TMR fluorescence of MHC was higher when labeled in relaxing solution than in rigor solution (Fig. 8A, 8B). This difference remained when the myofibril samples were labeled to saturation (Fig. S6). Changes in actin fluorescence were more variable. During brief labeling there was no difference in actin fluorescence between contractile conditions, although a second higher band often became visible (Fig. 8C), but saturation labeling did lead to increased actin fluorescence in rigor solution. The fluorescence values of the other myofilament proteins were not measurably different between conditions.

In ventricular myofibrils cTnC was the only myofilament protein whose cysteine accessibility was dependent on calcium (Fig. 8C–E). When myofibrils were briefly labeled in rigor or relaxing solutions there was primarily one upper cTnC band in the TMR scan, but in solutions with added calcium (independent of the presence of ATP) there was a second cTnC band (Fig. 8C and 8D). Prolonged labeling in a calcium solution led to a single upper band, indicating that one of cTnC’s two cysteines had not been completely inaccessible (Fig. 8D). Note that in the Sypro scan the two TnC bands and RLC have the same electorphoretic mobilities as also seen in Figures 5B and 7. To further analyze the effect of calcium on cTnC cysteine accessibility, ventricular myofibrils were exposed to different concentrations of calcium in rigor solution. In the gels the intensity of the lower and upper cTnC bands were modulated by [Ca^2+^] (Fig. 8E). The fractional increase of the lower cTnC band over a range of [Ca^2+^] had a pCa_50_ of 6.3, similar to pCa_50_ values recorded from ATPase assays (see below).

### III. Results of Experiments to Test Myofibril ATPase after Oxidant Treatments

**Figure 9:**
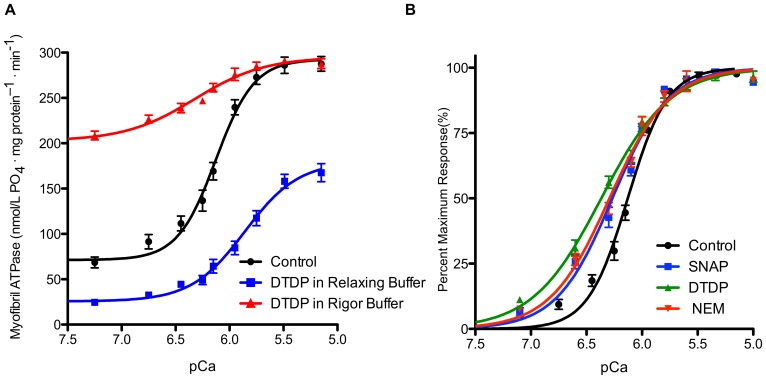
ATPase depends on the contractile state in which myofibrils are exposed to oxidants. **A**. ATPase-pCa relationships of rat ventricular myofibrils treated with DTDP (150 µmol/L DTDP) in relaxing or rigor solutions, or untreated (Control). Curves fit to a Hill equation. Values are mean ± SEM; *n* = 4. Myofibrils treated with DTDP in rigor solution had a markedly higher basal ATPase and significantly higher pCa_50_ than control values, but maximum ATPase rates were not significantly different from controls. Myofibrils treated with DTDP in relaxing solution had a markedly lower maximum ATPase. **B**. Normalized ATPase-pCa relationships from control myofibrils and myofibrils treated in activating solution with 75 µmol/L DTDP, 75 µmol/L NEM, or 2 mmol/L SNAP an NO Donor (mean ± SEM; *n* = 4). NEM induced changes in calcium sensitivity indicating that the changes in ATPase from DTDP treatment were not due to the formation of a disulfide bond. SNAP: the increased calcium sensitivity and basal ATPase from SNAP were due to the modification of cysteines.

ATPase depends on the contractile state in which myofibrils are exposed to oxidants. To test for functional effects that corresponded to changes in cysteine accessibility, we compared ventricular myofibril ATPases after exposure to the cysteine oxidant DTDP in rigor solution and in relaxing solution (Table 1). Myofibrils treated with DTDP in rigor solution had a markedly higher basal ATPase and significantly higher pCa_50_ than control values, but maximum ATPase rates were not significantly different from controls. In contrast, myofibrils treated with DTDP in relaxing solution had a markedly lower maximum ATPase. Cooperativity (*n*_H_) was decreased by DTDP treatment in both rigor and relaxing solutions, but was more affected by treatment in rigor solution. Increasing the concentration of [DTDP] (150 µmol/L instead of 75 µmol/L) caused an even larger disparity between effects - basal ATPase was even higher in rigor but lower in relaxing solution, maximal ATPase was even lower in relaxing and even closer to control in rigor solution, and calcium sensitivity (pCa_50_ ) was even higher in rigor but lower in relaxing solution (Table 1, Fig. 9A). Thus, oxidant treatments in rigor and relaxing solutions had opposite effects on myofibril ATPase rates and calcium sensitivity.

**Table 1:** Myofibril ATPase Parameters.

Treatment	Basal ATPase (nmol/LPO_4_ · mg protein^–1^ · min^−1^)	Maximum ATPase(nmol/L PO_4_ · mg protein^–1^ · min^−1^)	pCa_50_	*n*_H_
Ventricular Control	70.7±5.0	292.7±5.8	6.131±0.021	2.46±0.21
75 µmol/L DTDP Relax	97.2±4.2*	235.7±7.8*	6.066±0.017	1.78±0.14*
150 µmol/L DTDP Relax	25.9±2.5*	179.8±6.0*	5.860±0.019*	1.84±0.16
75 µmol/L DTDP Rigor	152.8±3.1*	275.6±7.3	6.281±0.053*	1.35±0.10*
150 µmol/L DTDP Rigor	202.0±4.6*	296.3±3.7	6.320±0.017*	1.38±0.17*
75 µmol/L DTDP Act	108.2±8.5*	237.9±8.6*	6.368±0.027*	1.43±0.12*
75 µmol/L NEM Act	56.8±2.9	199.0±4.7*	6.291±0.028*	1.64±0.11*
2 mmol/L SNAP Act	75.5±4.9	244.2±7.4*	6.270±0.032*	1.78±0.18*
Fast Twitch Control	123.9±2.8	349.5±6.4	6.155±0.017	2.85±0.21
75 µmol/L NEM Act	73.5±3.0*	190.1±8.8*	6.075±0.005*	2.80±0.21
75 µmol/L NEM Rigor	140.2±5.1*	328.5±6.3*	6.155±0.022	2.12±0.10*
150 µmol/L NEM Rigor	152.6±5.6*	327.3±11.6	6.208±0.033	2.20±0.14

All values are given as means ± SEM (*n* = 4).

* P<0.05 vs. control.

Next we tested whether treatment of myofibrils in a solution with ATP and calcium, which results in cross-bridges cycling through multiple attached and detached states, would cause a combination of affects seen in rigor and relaxing solutions. We found that myofibrils treated with DTDP in activating solution (75 µmol/L DTDP, 3 mmol/L ATP, pCa 5.0) had a reduced maximal ATPase similar to treatment in relaxing solution, and an increased pCa_50_ and *n*_H_ similar to treatment in rigor solution (Table 1).

To test whether the changes in ATPase depended on the formation of protein disulfides, we treated myofibrils with NEM, an oxidant similar to DTDP, but which does not produce protein disulfides. Treatment with NEM in activating solution, which allowed simultaneous measurement of effects found in rigor and relaxing solutions, decreased maximum ATPase, increased pCa_50_, and decreased *n*_H_ (Table 1, Fig. 9B). Thus, the changes in ATPase from DTDP treatment were not due to the formation of a disulfide bond.

Both NEM and DTDP are non-physiological reagents specific for cysteines. To test a naturally occurring oxidant, we used the NO donor SNAP. When ventricular myofibrils were exposed under activating conditions to 2 mmol/L SNAP (estimated to liberate a maximum NO concentration of 2.1 µmol/L [35], basal ATPase was somewhat elevated, maximum ATPase was significantly lower, pCa_50_ was higher and *n*_H_ was lower than controls (Table 1, Fig. 9B). These effects were similar to NEM and DTDP treatments, suggesting that the increased calcium sensitivity and basal ATPase from SNAP were due to the modification of cysteines.

Fast twitch muscle differs in cysteine content from cardiac muscle and may differ in structure (Fig. 1A). Testing the effects of oxidants on fast twitch myofibrils, we found that NEM treatment in activating solution decreased the maximum ATPase similarly to what occurred in cardiac muscle, but the increase in pCa_50_ found in cardiac myofibrils was absent in fast twitch myofibrils (Table 1). Due to the lack of effect on pCa_50_ in fast twitch myofibrils, we performed additional NEM treatments in rigor solution with higher NEM concentrations. Yet, the maximum ATPase of fast twitch myofibrils was only marginally decreased from treatment in rigor solution, and the pCa_50_ and basal ATPase were only marginally increased at the higher oxidant concentration. Thus, in both cardiac and fast twitch myofibrils the maximum ATPase decreased from oxidant treatment in activation solution, but the two muscle types differed in their capacity for oxidants to activate the thin filament.

## Discussion

Here we have used evolutionary, structural, and biochemical approaches to assess how cysteine oxidation modifies contractile function. We established the number of myofilament cysteines in different muscle types, and tracked their emergence across vertebrate evolution. To filter these cysteines, we assessed their accessibility to solvents in a series of crystal structures, which suggested that not all myofilament cysteines would be accessible to oxidants and that mixing troponin isoforms may lead to disulfide formation. To validate the accessibility measures, we treated myofibrils with a fluorescent cysteine probe and visualized accessibility using gel electrophoresis. We demonstrate that not all myofilament cysteines are accessible in the myofilament lattice, and that the contractile state in which myofibrils are exposed to oxidants affects cysteine accessibility and ATPase activity. Oxidant exposure to ventricular and fast twitch myofibrils under relaxing conditions exposes an MHC cysteine, and this modification corresponds to a decrease in maximum ATPase, an effect that is absent in myofibrils exposed to oxidant in rigor solution. Oxidant exposure under rigor conditions produces modifications that increase basal ATPase and calcium sensitivity. These changes occurred in ventricular myofibrils, but were muted in fast twitch muscle. In summary, these experiments reveal how structural and sequence variations create divergent oxidative effects in striated muscle.

Over the course of animal evolution isoforms for multiple myofilament proteins arose. We found that in proteins with two or three isoforms the majority of cysteines, but not all, have been conserved across vertebrate evolution (Fig. 1 and 2). As a consequence, there are shared cysteines because the same isoform is commonly expressed, and shared cysteines because the cysteine has been conserved in a second isoform. By pooling all of the myofilament proteins, we established the similarities in cysteine content between fiber types. This suggested that ventricular and slow twitch muscle would have similar responses to oxidant exposure due to the repeated co-expression of the same isoform. These analyses also showed, assuming minimal structural differences between isoforms, only a few cysteines may explain how oxidants affect muscle types differently. One of those cysteines (Cys 134) is found in fast TnI, and was recently suggested to increase calcium sensitivity when glutathionylated [39], a result that illustrates how unique cysteines can produce fiber type specific effects. Other unique cysteines (Fig. 2) are found in the neck region of fast MHC (Cys 796, Cys 817), cTnC (Cys 35, Cys 84), TnI, and the two light chains (fRLC C126, C157; cMLC1 C81). Interestingly, two unique cysteines, one found in fast muscle (TnC Cys 99), and the other expressed in slow muscle (TnI Cys 87) if present in the same complex would be located approximately 4 angstroms apart and within range to form a disulfide bond (Figure 3). This has potential implications on the effects of ‘mixed’ troponin complexes in fibers.

### New Methods to Evaluate Cysteine Accessibility

ROS modulation requires ROS accessibility. To filter cysteines that may be functionally important, we estimated the accessibility of cysteines to solvent using RSA values calculated from protein crystal structures. We also assessed cysteine modifications in the myofilament lattice by treating myofibrils with a fluorescent reagent specific for cysteines (TMR). The use of a cysteine specific fluorescent reagent overcomes several limitations present in other methods including Ellman’s reagent, immunoblotting, and mass spectroscopy. Recording the total thiol status with Ellman’s reagent fails to indicate which proteins have modified cysteines, and the final output is dependent on the relative expression of each protein. Immunoblots can identify bands with modified cysteines, but are limited by the antibody’s ability to bind all proteins with cysteine modifications, but not bind proteins where cysteines have not been modified. Mass spectroscopy is the gold standard for identifying posttranslational modifications, but for our purposes became limiting when trying to sample all myofilament peptides, and the associated cost of testing multiple conditions and fiber types. Due to these constraints, we developed a TMR based method, which does not require the additional steps of an immunoblot, it directly modifies proteins in their native state and therefore does not have the limitations of an antibody recognizing a unique epitope, it has high specificity for cysteines, and it can reveal the number of modified cysteines in a protein. Furthermore, it is cost effective to assess a range of treatments, and its fluorescence is photostable, pH insensitive, and has a large dynamic range.

Previous experiments have found that every cardiac myofilament protein, except TnT and RLC, has a cysteine that can be modified by oxidants [14]–[17]. Combining our analyses of the primary amino acid sequences (Fig. 1), RSA values from protein structures (Fig. 3), and cysteine labeling in myofibrils [Figs. 4, 5, 6, and 7], we too found that nearly all skeletal and cardiac myofilament proteins have accessible cysteines. These data on cysteine accessibility match previous experiments on isolated proteins, and expand the number of analyzed proteins and their accessibility in the myofilament lattice.

We found that the cardiac and fast skeletal TnC isoforms differed in their cysteine contents and accessibilities. The cysteine in fast skeletal muscle was not accessible for TMR labeling, but the two cysteines in cTnC were accessible. The accessibilities of the cTnC cysteines depended on the presence of calcium, and somewhat on the presence of ATP. These dependences on calcium are consistent with investigations using the isolated troponin complex that found Cys 35 to be less reactive in the presence of calcium [40], and Cys 84 to be more reactive in the presence of calcium [41], [42].

Of the three fTnI cysteines, Cys134 is highly accessible based on RSA data, but the other two cysteines were only marginally accessible. This matches data on the isolated troponin complex, in which only one of the fTnI cysteines was found accessible [42]–[44]. In cTnI we found only marginal cysteine accessibility, consistent with the RSA values and the modest accessibility of the two cysteines common to fTnI. We speculate that sTnI has two accessible cysteines (Figure 6B), but there is no published structure and we were unable to study the slow isoform in isolation, because fast and slow isoforms are co-expressed.

Our fluorescence data suggest that only one or two of actin’s five cysteines are accessible, and RSA data suggests that Cys 374 and Cys 10 are the most likely candidates. Our data also suggests that accessibility increases in rigor solution compared to relaxing solution. Previously, skeletal muscle actin has been measured to have one accessible cysteine (Cys 374), with accessibility to a second site (Cys 10) occurring in a different contractile state [45]. Also in the thin filament, Cys 190 of Tm has been reported to form a disulfide with the equivalent cysteine in the second protein of the Tm dimer [46]; we also found this cysteine accessible.

The fast isoform of RLC contains two accessible cysteines, as indicated by the double bands in the PAGE gels (Figs. 4, 5, and 6), and this matches data on purified myosin [47], [48]. In fast isoforms of MLC we find two accessible cysteines, but only one accessible cysteine in the ventricular MLC1 isoform (Fig. 7). Based on the single cysteine common to cardiac and fast isoforms, the accessible cysteine in the ventricular isoform is probably Cys 187, and the inaccessible cysteine is probably Cys 81. Fast MHC contains 16 cysteines, 10 of which are located in the S1 segment. In S1, 6–10 cysteines are accessible, depending on the conditions of measurement [49]–[51]. We found a slight majority of MHC’s cysteines to be accessible, based on comparison between fluorescence when myofibrils were labeled in relaxing or urea solutions.

### Modifications in Myofibril Cysteine Accessibility Depends on ATP Presence during Oxidant Exposure

By altering the contractile state it is possible to separate out which cysteines affect function if there are corresponding changes between function and cysteine accessibility. We found that the accessibility of cysteines in myosin, cTnC, and actin were dependent on the contractile state and thus suggest mechanisms for changes in function. We found no other proteins with large changes in fluorescence. This therefore rules out a large segment of the cysteine population for having functional effects that are dependent on the contractile state. We found that MHC had fewer accessible cysteines, and actin had more accessible cysteines in rigor solution than in relaxing solution. These data are consistent with experiments on fast muscle fibers where treatment with NEM in rigor solution produced fewer MHC modifications and more actin modifications compared to treatment in relaxing solutions [45]. Additionally, in a more detailed analysis, it has been found that treatment of frog skeletal muscle with NEM led to four MHC cysteines becoming less reactive in contracting muscle compared to relaxed muscle, while one new cysteine became accessible [52], [53]. Using our methods we can only measure net changes in cysteine accessibility and thus would be unable to quantify a newly accessible cysteine, yet we believe this finding has an important bearing on our ATPase data.

### Modifications in Myofibril ATPase also Depends on ATP Presence during Oxidant Exposure

Corresponding to the changes in cysteine accessibility, we found different effects on ATPase rates when ventricular myofibrils were exposed to oxidants in different contractile states. Treatment in relaxing solution depressed ATPase, pCa_50_ and cooperativity. In contrast, treatment in rigor solution had no effect on maximum ATPase, but increased the basal ATPase and pCa_50_. Treatment in activating solution, where myosin heads are cycling through attached and detached structural states, produced a combination of effects found from treatment in relaxing and rigor solutions. The changes in ATPase were present with two other oxidants (NEM, NO) suggesting the changes in function were not unique to one oxidant and that protein disulfide bonds were not responsible for the oxidant effects. These functional effects in the myofibril preparation are consistent with effects measured in muscle fibers: human cardiac fibers treated in relaxing solutions with DTDP had qualitatively similar reductions in pCa_50_ [16], and experiments conducted with slow muscle fibers in which sarcomere lengths were maintained found increases in pCa_50_ similar to the increase in pCa_50_ that we observed in myofibrils [29].

The decrease in maximum ATPase from oxidant treatment in relaxing solution corresponded to changes in MHC cysteine oxidation. No other protein had an increase in gel fluorescence from treatment in relaxing solution compared to rigor solution. This suggests that an MHC cysteine critical to ATP hydrolysis was accessible in relaxing solution, but inaccessible in rigor solution where maximum ATPase was protected. Cysteines from other proteins were modified in relaxing solution and could produce additional functional effects, but these effects would be independent of the muscle’s contractile state. Of MHC’s 14 cysteines, the cysteine most likely responsible is cysteine 706 (Rat MYH6), also known as SH1. In fast skeletal muscle this site is more reactive in relaxing conditions [45], and its modification inhibits myosin binding to actin and consequently actin activated myosin ATPase [54]. Additionally, modification to this site would modestly depress calcium sensitivity, as we found, by reducing the number of strongly bound cross bridges, since strongly bound cross-bridges increase the binding of calcium by TnC [55].

The effects from oxidant treatment in rigor solution were different from relaxing solution, and were distinguished by activation of the thin filament (increased basal ATPase and pCa_50_). These effects were significant in cardiac myofibrils, but were muted in fast twitch myofibrils. Oxidants could activate the thin filament if modification of an MHC cysteine led to a population of rigor cross-bridges remaining after oxidant treatment. These strongly bound cross bridges could alter the thin filament regulatory structure allowing active cross bridges in the absence of calcium, and increased cooperativity within and between regulatory units in the presence of calcium. The thin filament could also be activated by modification of a cysteine in actin, TnI, or TnC. For instance, modification of a thin filament cysteine could lead to a structural state that kept the switch peptide of TnI bound to TnC, the inhibitory domain of TnI away from actin, or Tm partially in the open state. Each scenario could allow actin-myosin interactions in the absence of calcium.

One reason to suggest that an MHC cysteine may play a role in thin filament activation is that the effects we measured from oxidant treatments in rigor solution match effects from the addition of exogenous NEM-S1 to skinned rat myocardium [56], [57]. In these experiments, added NEM-S1 increased the number of strongly bound cross-bridges, which led to active force in the absence of calcium (∼20% of maximum active force) and an increased calcium sensitivity (pCaΔ = 0.11). Furthermore, the addition of NEM-S1 to fast twitch muscle fibers led to only a small increase in basal force (∼5%) and no change in pCa_50_. Thus, our results from oxidant treatment of cardiac and fast myofibrils match results from treatment of cardiac and fast fibers with myosin heads modified by oxidants. Furthermore, although we measured a decrease in the total number of accessible MHC cysteines in rigor conditions, based on prior literature [52], [53] at least one cysteine does become accessible.

The comparison of cysteine content and the dependence of contractile state on the effects from oxidants helps clarify previous data where similar and divergent effects were found between different muscle types and adds to the elegant work from the Lamb laboratory [28]–[30]. When *in vitro* preparations from cardiac, slow, or fast twitch muscle are in relaxing solution, adding ROS reduces the pCa_50_ and maximum force production [16], [29], [21]. On the other hand, if cardiac and slow muscle preparations are activated or in rigor when ROS is added calcium sensitivity increases, often without a loss of force or ATPase [29], [20], [24]. This increase in calcium sensitivity is absent in fast twitch muscle fibers [29], [51]. Together these data indicate that in relaxing solution the effects from oxidants are similar between cardiac, slow, and fast twitch muscle types, but in rigor or activating solution thin filament activation is unique to cardiac and slow muscle preparations.

Activation of the thin filament in cardiac and slow twitch but not in fast twitch muscle could be due to differences in cysteine content or differences in protein structure. Eight of the nine cysteines in the head region of MHC are conserved between the fast isoform (MYH4) and the cardiac isoform (MYH6), but the one unique cysteine in MYH6 (Cys 36) is not conserved in the slow twitch isoform (MYH7) indicating it is not responsible. This suggests that structural differences between fiber types rather then a unique cysteine is responsible for the absence of thin filament activation in fast twitch muscle. One key structural difference is found in TnC, which has two isoforms, one expressed in fast twitch muscle, and a second expressed in slow and cardiac muscle. These isoforms differ in structure and cysteine content. Furthermore, strongly bound cross bridges lead to further structural differences between TnC isoforms [58]. Thus, oxidation of a cysteine that promotes strongly bound cross bridges could affect TnC isoforms differently and therefore explain how oxidation leads to thin filament activation in slow and cardiac muscle but not in fast twitch muscle.

### Conclusions

We performed a thorough characterization of the cysteine content between different muscle types and vertebrate species. We then used a novel set of methods to discover that the accessibility of myofilament cysteines to oxidants differs between contractile states. We also showed that the effects of oxidation on ATPase activity depends on the contractile state in which myofibrils are exposed to oxidants: in cardiac myofibrils, changes in basal ATPase and pCa_50_ were consistent with oxidation of cysteines affecting thin filament activation, while changes in maximum ATPase were consistent with oxidation of a cysteine in MHC that is essential for ATP hydrolysis. Myofibrils from fast twitch skeletal muscle exposed to oxidants showed the same effect on maximum ATPase, but not on thin filament oxidation. Together these results link different effects found between cardiac, slow and fast twitch muscle, and provide new insight into the mechanisms by which skeletal and cardiac muscle are affected by oxidants, and the potential role of oxidant exposure in contractile function.

## Supporting Information

Figure S1 **Sequence comparisons of fast TnI between vertebrate species.** A. Conservation of histidine (green), methionine (blue) and cysteine (red) residues in the human sequence compared to other vertebrate species. Horizontal solid lines represent the position of an amino acid in the primary sequence of fTnI. Vertical dashed lines connect conserved amino acids. B. Evolution of Cys 134 from fTnI in vertebrates.
(TIFF)

Figure S2 **Sequence comparisons of slow and fast MLC isoforms in vertebrates.** Threonine 60 (rat fMLC1), a phosphorylation site conserved between isoforms [37], is highlighted in blue. Cysteine residues are highlighted in red. Cys 81 is conserved in the slow isoform across vertebrate evolution. Cys 63 is present in only a subset of fast and slow vertebrate species.
(TIFF)

Figure S3 **Fast isoforms of MHC contain two unique cysteines in the neck region.** A. Alignment of MHC isoforms expressed in cardiac (alpha, beta), slow (slow) and fast twitch (2X, 2A, 2B) muscle types. The three main structural domains of MHC are color coded (red = head, neck = blue, and tail =  green). Cysteines are marked by closed circles. B. Locations of the two neck cysteines and their relation to the two light chains in structures. The head region of MHC is red and the neck region is blue. The tail region is not included in the structures. C. Alignment of amino acid sequences of the neck cysteines from different MHC isoforms (IQ motifs are highlighted blue).
(TIFF)

Figure S4 **TMR has wide access to cysteines in the skeletal myofilament lattice.** **A&B**. TMR and Sypro scans of SDS PAGE gels from rat EDL myofibrils treated for five minutes with progressively greater concentrations of TMR in rigor solution.
(TIFF)

Figure S5 **TMR has wide access to cysteines in the cardiac myofilament lattice.** TMR scan of SDS PAGE gel from ventricular myofibrils treated for five minutes with progressively greater concentrations of TMR in rigor solution. To saturate TMR labeling and to label all cysteines, myofibrils were also treated in 750 µmol/L TMR for 60 minutes, or labeled in denaturing solution (right two lanes). A Sypro stain visualized total protein (furthest left lane) and RLC (below TMR scan). RLC has been used as a loading control, as RLC has no cysteines.
(TIFF)

Figure S6 **Saturation labeling of ventricular myofibrils reveals accessibility differences between rigor and relaxing solutions.** **A.** Scan of SDS PAGE gel of ventricular myofibrils labeled to saturation with TMR in rigor and relaxing solution. **B.** Plot of MHC and actin TMR fluorescence (arbitrary units = AU). * Denotes a p<0.05 for rigor fluorescence compared to relaxing fluorescence.
(TIFF)
