# Supplementary figures and images for: Accessibility of Myofilament Cysteines and Effects on ATPase Depend on the Activation State during Exposure to Oxidants
Source: PLoS One. 2013 Jul 19;8(7):e69110. doi: 10.1371/journal.pone.0069110 (PMC3716824; doi:10.1371/journal.pone.0069110)

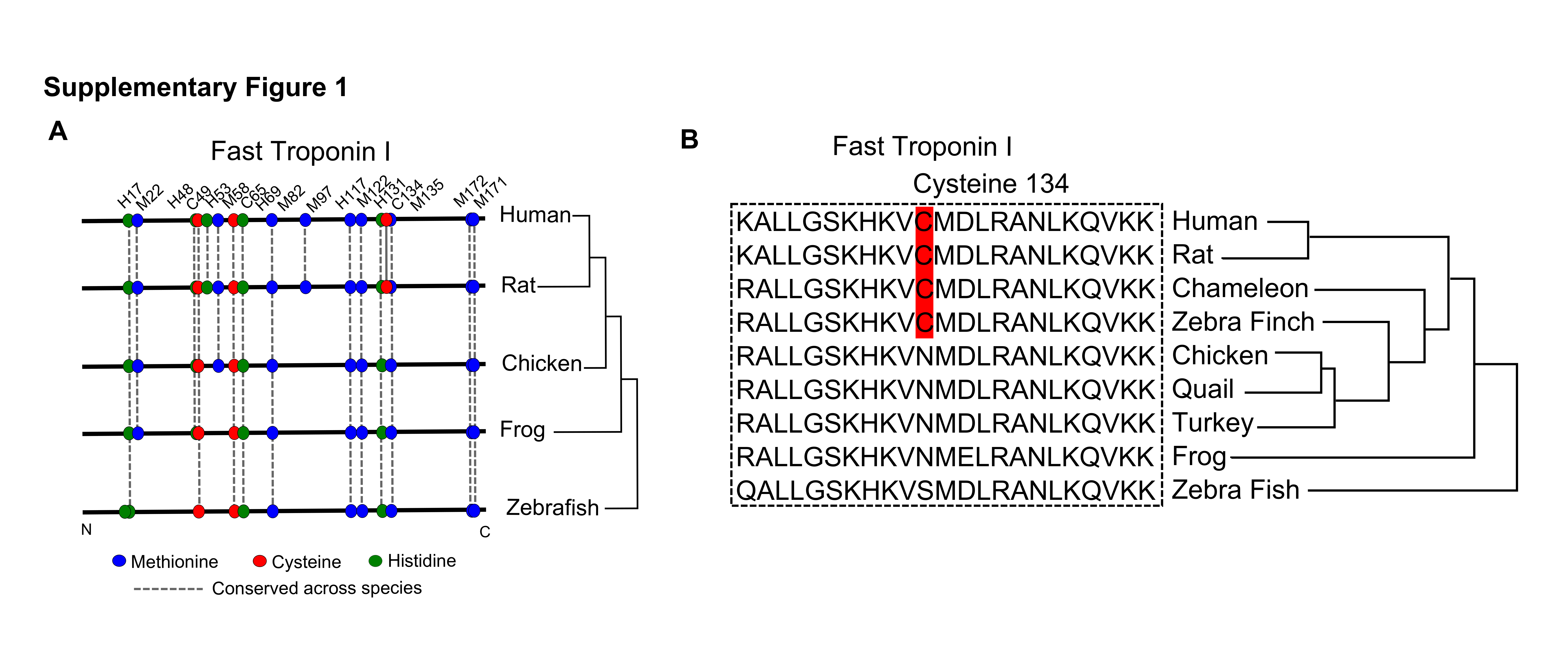

Supplement: Figure S1 — Sequence comparisons of fast TnI between vertebrate species. A. Conservation of histidine (green), methionine (blue) and cysteine (red) residues in the human sequence compared to other vertebrate species. Horizontal solid lines represent the position of an amino acid in the primary sequence of fTnI. Vertical dashed lines connect conserved amino acids. B. Evolution of Cys 134 from fTnI in vertebrates. (TIFF) [file pone.0069110.s001.tiff]

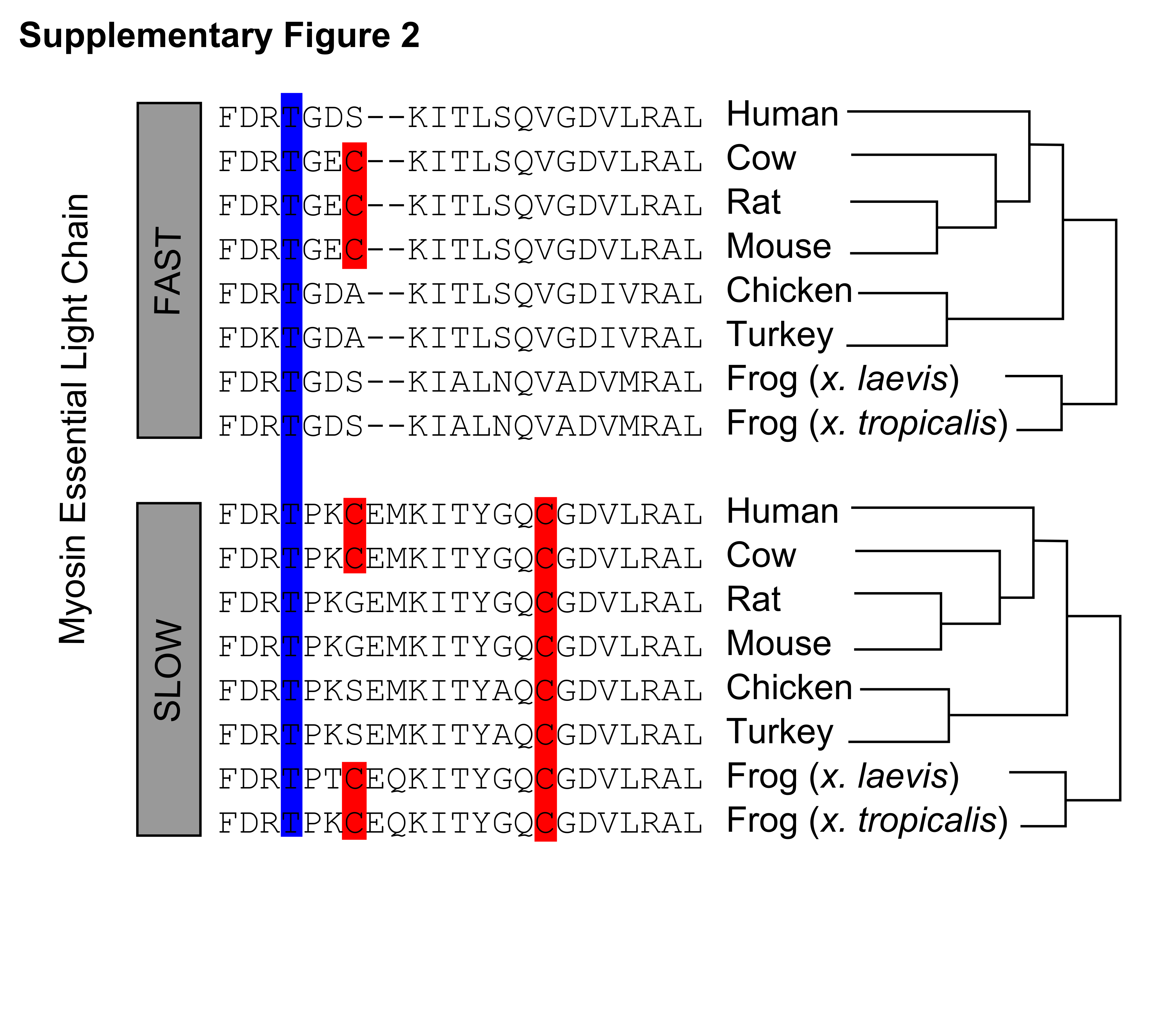

Supplement: Figure S2 — Sequence comparisons of slow and fast MLC isoforms in vertebrates. Threonine 60 (rat fMLC1), a phosphorylation site conserved between isoforms [37], is highlighted in blue. Cysteine residues are highlighted in red. Cys 81 is conserved in the slow isoform across vertebrate evolution. Cys 63 is present in only a subset of fast and slow vertebrate species. (TIFF) [file pone.0069110.s002.tiff]

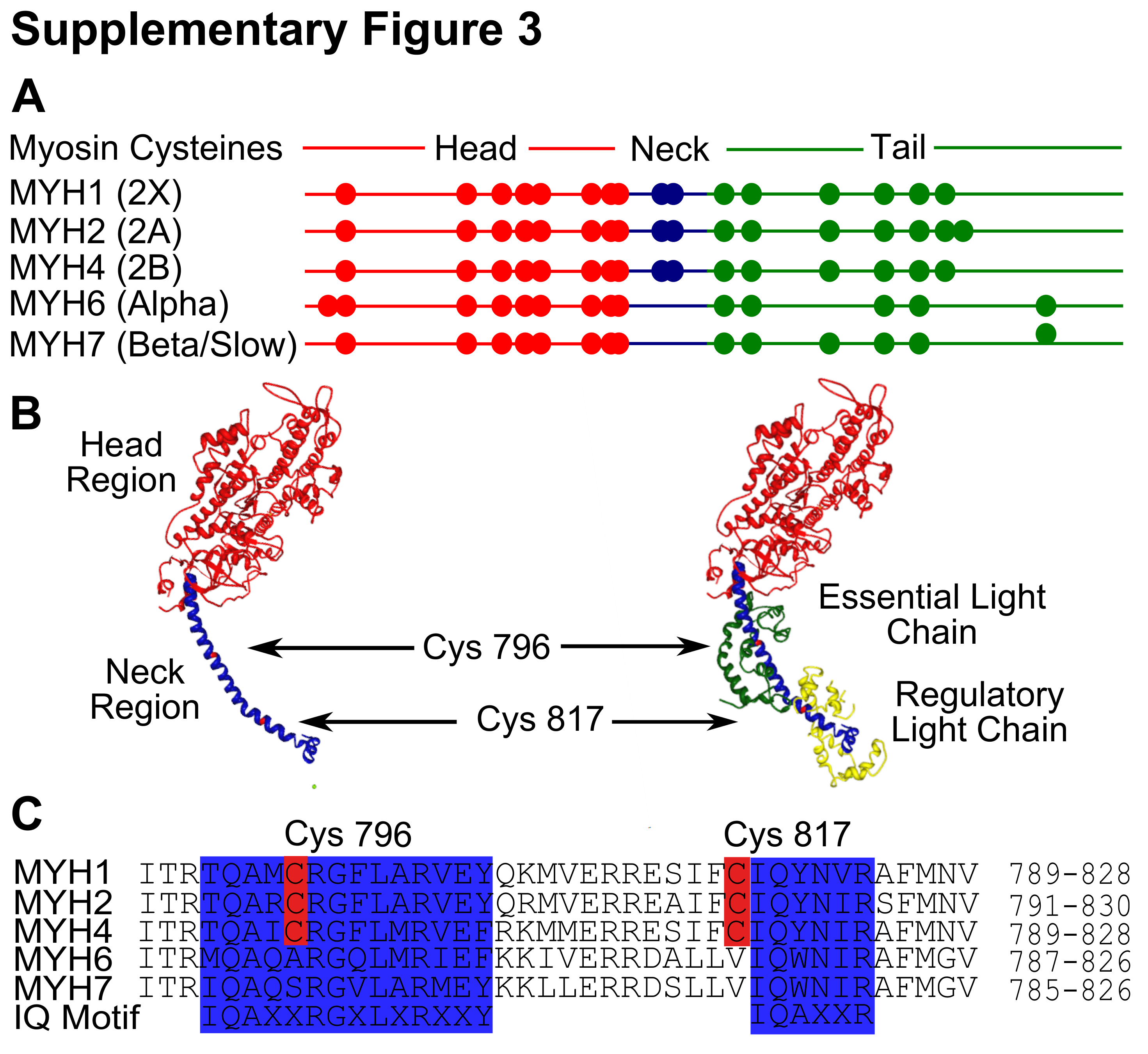

Supplement: Figure S3 — Fast isoforms of MHC contain two unique cysteines in the neck region. A. Alignment of MHC isoforms expressed in cardiac (alpha, beta), slow (slow) and fast twitch (2X, 2A, 2B) muscle types. The three main structural domains of MHC are color coded (red = head, neck = blue, and tail = green). Cysteines are marked by closed circles. B. Locations of the two neck cysteines and their relation to the two light chains in structures. The head region of MHC is red and the neck region is blue. The tail region is not included in the structures. C. Alignment of amino acid sequences of the neck cysteines from different MHC isoforms (IQ motifs are highlighted blue). (TIFF) [file pone.0069110.s003.tiff]

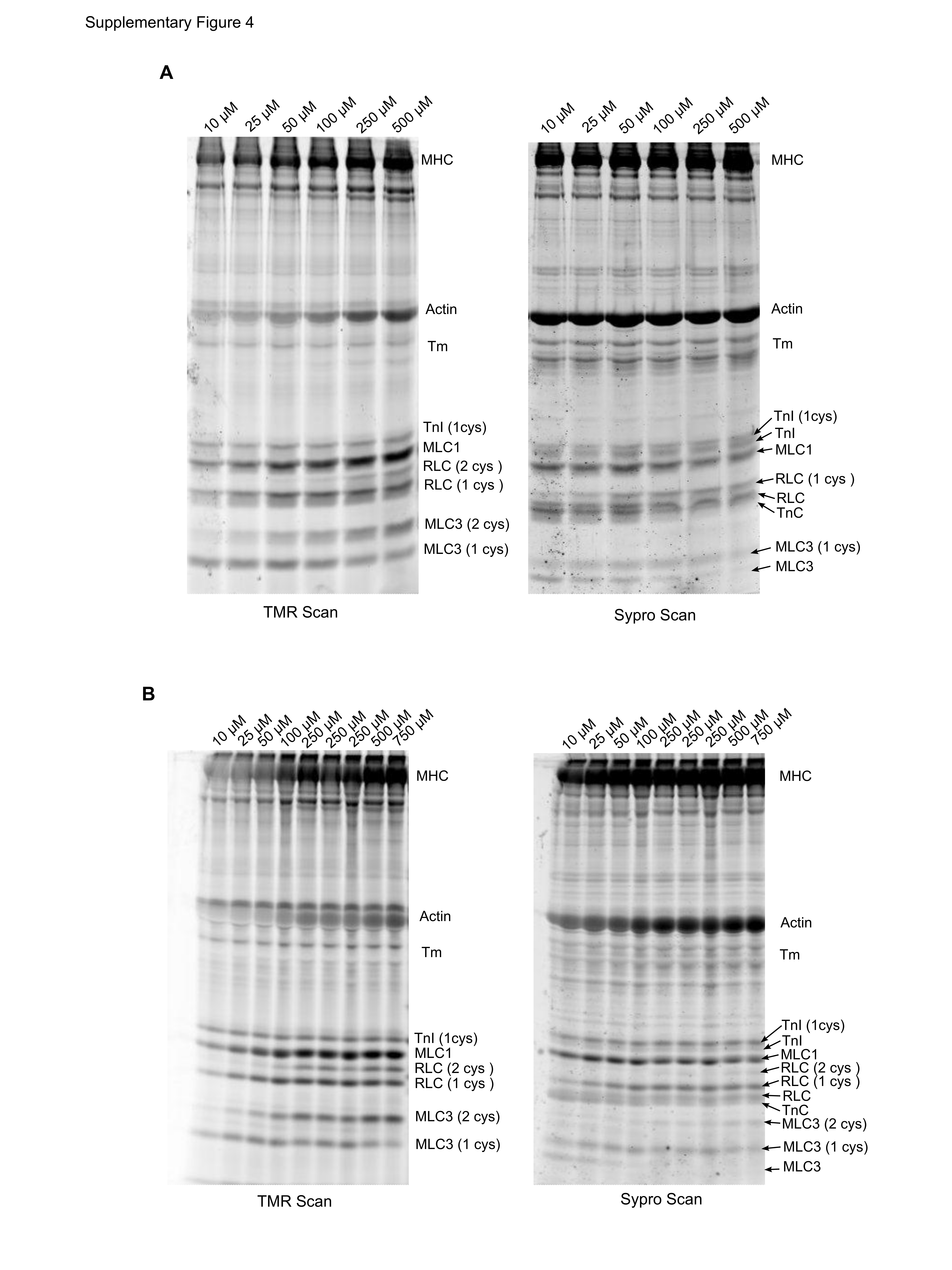

Supplement: Figure S4 — TMR has wide access to cysteines in the skeletal myofilament lattice. A&B. TMR and Sypro scans of SDS PAGE gels from rat EDL myofibrils treated for five minutes with progressively greater concentrations of TMR in rigor solution. (TIFF) [file pone.0069110.s004.tiff]

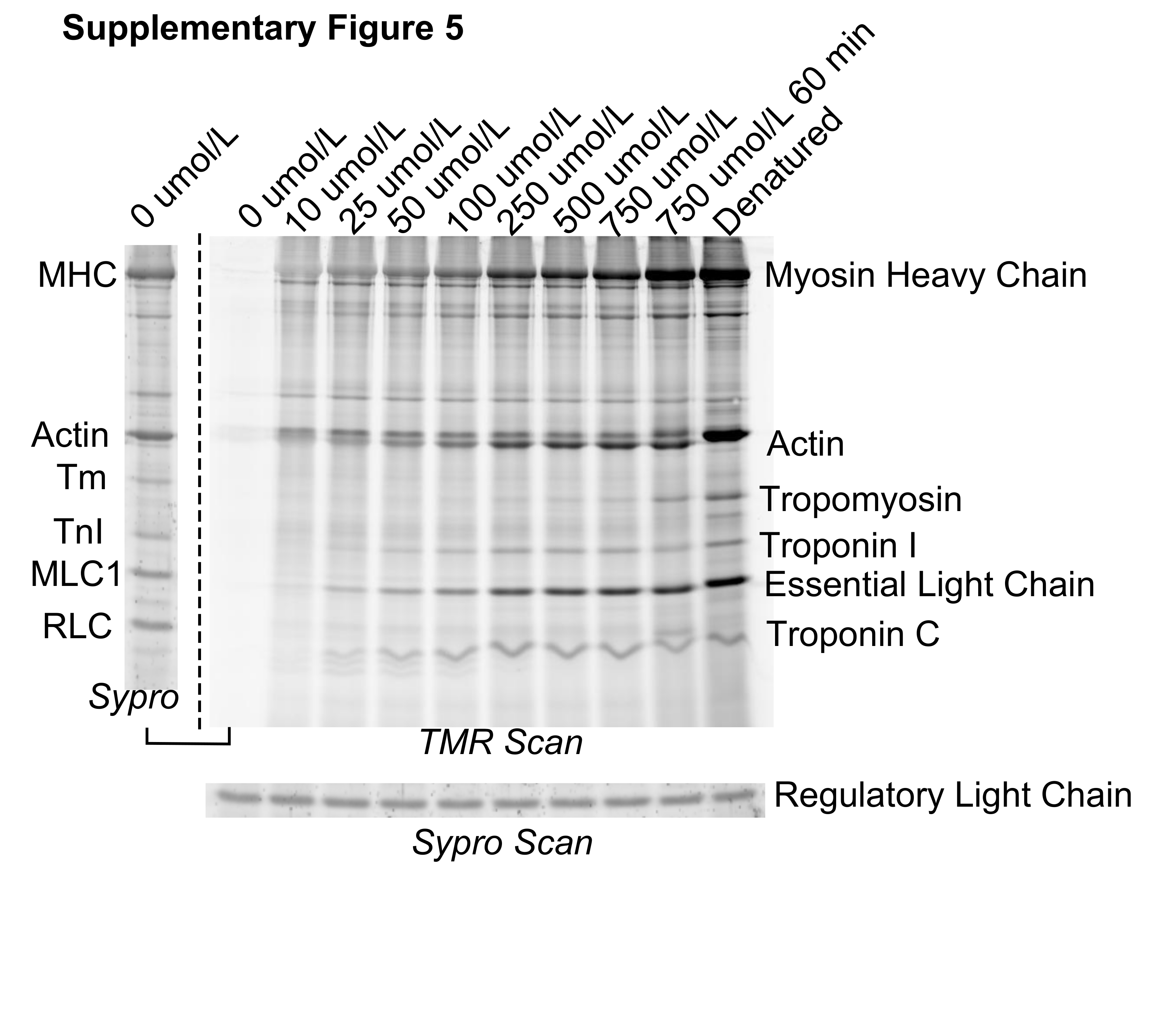

Supplement: Figure S5 — TMR has wide access to cysteines in the cardiac myofilament lattice. TMR scan of SDS PAGE gel from ventricular myofibrils treated for five minutes with progressively greater concentrations of TMR in rigor solution. To saturate TMR labeling and to label all cysteines, myofibrils were also treated in 750 µmol/L TMR for 60 minutes, or labeled in denaturing solution (right two lanes). A Sypro stain visualized total protein (furthest left lane) and RLC (below TMR scan). RLC has been used as a loading control, as RLC has no cysteines. (TIFF) [file pone.0069110.s005.tiff]

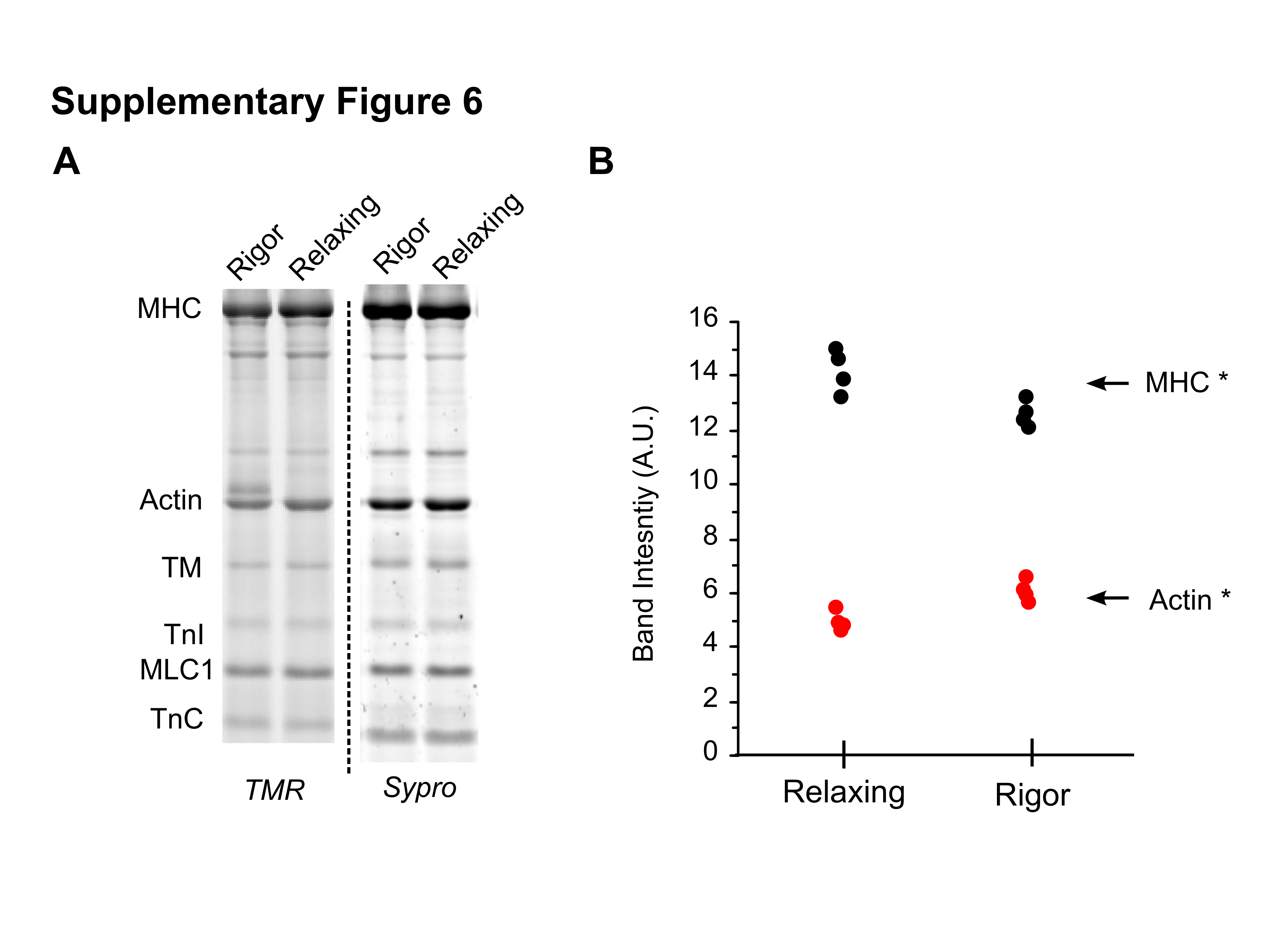

Supplement: Figure S6 — Saturation labeling of ventricular myofibrils reveals accessibility differences between rigor and relaxing solutions. A. Scan of SDS PAGE gel of ventricular myofibrils labeled to saturation with TMR in rigor and relaxing solution. B. Plot of MHC and actin TMR fluorescence (arbitrary units = AU). * Denotes a p<0.05 for rigor fluorescence compared to relaxing fluorescence. (TIFF) [file pone.0069110.s006.tiff]
